# Skin Displacement as fascia tissue manipulation at the lower back affects instantaneously the flexion-and extension spine, pelvis, and hip range of motion

**DOI:** 10.3389/fphys.2022.1067816

**Published:** 2022-11-23

**Authors:** Robbert N. van Amstel, Richard T. Jaspers, Annelies L. Pool-Goudzwaard

**Affiliations:** ^1^ Department of Human Movement Sciences, Faculty of Behavioural and Movement Sciences, Amsterdam Movement Sciences, Vrije Universiteit Amsterdam, Amsterdam, Netherlands; ^2^ Fysio Science Department, Fysio Physics Group, IJsselstein, Netherlands; ^3^ SOMT, University of Physiotherapy, Amersfoort, Netherlands

**Keywords:** biomechanics, spine, pelvis, hip, range of motion, fascia

## Abstract

Low back pain (LBP), associated with spine, pelvis, and hip mobility impairments can be caused by tight muscle contractions, to protect sensitized lumbar fasciae. Fascia tissue manipulations are used to treat lumbar fascia in LBP. The effect of fascia tissue manipulations through lumbodorsal skin displacement (SKD) on mobility is inconclusive likely depending on the location and displacement direction of the manipulation. This study aimed to assess whether lumbodorsal SKD affects the flexion -and extension range of motion (ROM), in healthy subjects. Furthermore, we aimed to test the effect of SKD at different locations and directions. Finally, to assess intertester and intratester reliability of SKD. Effects of SKD were tested in a motion capture, single-blinded, longitudinal, experimental study. Sixty-three subjects were randomly assigned to SKD- or sham group. SKD group was subjected to either mediolateral directed SKD during flexion or extension movement, *versus* a sham. The thoracic, lumbar, and hip angles and finger floor distance were measured to assess the change in ROM. Statistics indicated that the effect size in instantaneously change of flexion -and extension ROM by SKD was large (Effect size: flexion η^2^ *_p_* = 0.12–0.90; extension η^2^ *_p_* = 0.29–0.42). No significant effect was present in the sham condition. Flexion ROM decreased whereas the extension ROM increased, depending on SKD location- and displacement direction (*p* < 0.05). The ICC indicates a good intertester and intratester reliability (resp. ICC_3,k_ = 0.81–0.93; ICC_3,1_ = 0.70–0.84). Lumbodorsal SKD affects the flexion- and extension spine, pelvis, and hip range of motion. The effects of SKD are direction- and location dependent as well as movement (flexion/extension) specific. Lumbodorsal SKD during flexion and extension may be useful to determine whether or not a patient would benefit from fascia tissue manipulations. Further research is required to obtain insight into the mechanisms *via* which the SKD affects ROM and muscle activation, in healthy, asymptomatic-LBP, and LBP subjects.

## Introduction

Low back pain is associated with spine, pelvis, and hip mobility impairments (Reis and Macedo, 2015; Nishimura and Miyachi, 2020) hypothesized to be caused by tight muscle contractions to protect sensitized lumbar tissues (Hodges and Tucker, 2011; Van Dieën et al., 2019). Fasciae are specialized connective tissue structures and exist of various phenotypes like the superficial fascia, deep fascia, myofascia, and arthrofascia (joint capsules and ligaments). Each fascia (single connective tissue sheet incl. expansions) has an important role in transmitting force toward muscles and bones in a three-dimensional fashion (Huijing, 2002; Maas, 2019). Pathophysiological lumbar fasciae adaptations (Langevin et al., 2011; Tesarz et al., 2011) can influence this force transmission resulting in painful asymmetric muscle contraction (Kim et al., 2013; van Dieën et al., 2017) and loss in joint mobility (Maas, 2019). Both painful muscle contraction and loss of mobility are treatment parameters (Staal et al., 2017; Shipton, 2018).

In physical therapy, “fascia tissue manipulation(s)” (FTM(s) are used in treating musculoskeletal pain, like low back pain. FTMs such as myofascial release techniques, myofascial trigger-point interventions, and elastic tape application methods have been applied and their effectiveness have been systematically reviewed (Liu et al., 2019; Zhang et al., 2019; Arumugam and Harikesavan, 2020). The effectiveness of lumbar FTMs have been demonstrated regarding pain relief and improvement of joint mobility (Vanti et al., 2015; Wu et al., 2021). However, the effects of lumbar FTMs do not unequivocally prove to be successful (Chen et al., 2021; van Amstel et al., 2021). The explanation for the inconclusive results can be the differences in type, intensity, location, and/or direction of the utilized lumbar FTM in the above-mentioned studies.

Optimization of FTMs requires a more detailed understanding of the underlying mechanisms of this type of treatment. It has been proposed that FTMs by displacement of the skin, the tension in the underlying fasciae will be modulated which alters the mechanical properties. In support of this rationale, mathematical geometric modeling has shown that forces exerted onto the skin can deform and displace the fasciae and as such change the mechanical properties of the underlying fasciae (Chaudhry et al., 2008; Chaudhry et al., 2014). As evidence for these working mechanisms is lacking, several theoretical models have been proposed.

Regarding the effectiveness of FTMs on pain and mobility, it has been proposed that this will depend on both location and direction of the applied skin displacement (SKD) (Noten, 2021). It has been proposed that SKD will affect fasciae stiffness and their relative positions to surrounding tissues (Huijing and Baan, 2003; Maas, 2019), which can be beneficial but may also “worsen” pain and decrease mobility (Noten, 2021). To indicate whether or not a patient would benefit from FTMs, a fascial diagnostic test has been proposed: The Dynamic ArthroMyofascial Translation^®^ Test. The test consists of 3 steps: 1) affirmation of the most painful movement from stance to either flexion or extension, as a reference test, 2) the same test with ongoing mediolateral directed SKD to the right at e.g., L3 or L5, and 3) the same reference test with ongoing SKD to the left. SKD leading to the largest mobility improvement and/or pain reduction can be utilized for FTMs at the tested location (Noten, 2021).

Several studies in which FTMs have been applied to healthy humans by elastic tape or myofascial release have shown that fasciae and muscles below the skin undergo deformations and are locally strained (Tu et al., 2016; Wong et al., 2017; de las Penas, 2019; Wang et al., 2019). Therefore, it is conceivable that variable effects in alterations in mobility (i.e., increase or decrease) due to FTMs by SKD are also expected to occur in healthy subjects, but could be less pronounced than in patients with limited mobility for instance in case of low back pain.

Although changes in joint mobility through SKD seem to be clinically effective, the basal effects of SKD on healthy subjects have not been tested objectively. Therefore, the aims of this study were: 1) to assess whether SKD at the lower back affects flexion- and extension range of motion of the spine, pelvis, and hip complex *versus* a sham skin-displacement, in healthy subjects, and 2) if present, to test the effects of SKD at different locations and directions, as well as 3) to assess intertester and intratester reliability of applying the SKD.

## Materials and methods

Participants were recruited from the student and employee population of the Vrije Universiteit Amsterdam using posters and flyers and *via* advertisements that were placed on social media platforms (Facebook and Linkedin). Inclusion criteria were: Healthy subjects (BMI range between 18.5 < 30, age 25 till 55 years, able to read and speak English). We have chosen this age category because disability due to low back pain is highest or most severe at the age of 25–65-year (Hartvigsen et al., 2018). Exclusion criteria were low back pain within the last 6 months and other injuries.

### Priori power analysis

*A priori* power analysis for repeated measure ANOVA-Mix design (Gpower©program) was performed. The following values were used for the expected effects of SKD: 1-β = 0.80, α = 0.50, effect size f2 = 0.15, 2*4, resulting in a minimum of 56 participants, $F_{critical}$ = 2.41. The small effect size is based on clinical experience. Ten percent was added to these 56 participants (*n* = 60) for possible dropouts, in line with the COSMIN (Terwee et al., 2007; Mokkink et al., 2012).

### Randomization

Two experimental “fascial diagnostic test” (FDT) groups were created: an SKD- and a sham group. The stratified randomization method was used to secure homogeneity between the groups with regard to sex. The randomization was performed by a “blinded” observer, utilizing a computer-generated randomized table. Furthermore, each subject was randomly assigned to one of the four pre-selected orders of testing.

### Motion capture

**FIGURE 1:**
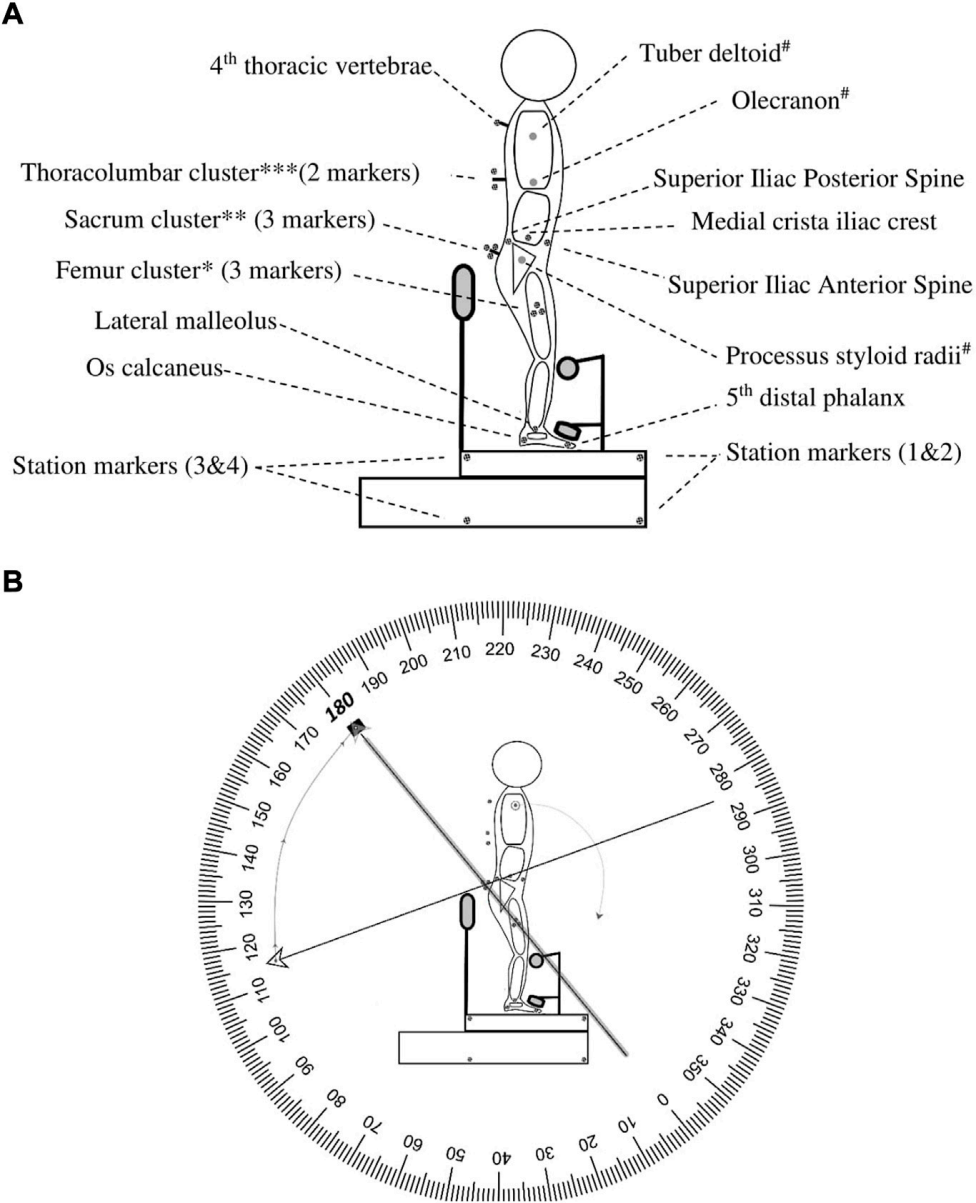
Marker placement and angle interpretation. This figure represents the marker placements for motion capture. **(A)**. *Femur cluster: represents femur, middle point between trochanter—lateral femoral condyle (measured with tape measure); **Sacrum cluster: displays S1, S2, and S3 (placed on S2/3); ***Thoracolumbar Cluster: Displays T12 -T8 (placed on T9), # is the arm which is not represented. **(B)**. Example HROM: When the terminal (←) and initial (▪) vectors were collinear it was defined as an angle of 180 degrees. In this example, a thoracic, lumbar, and hip flexion is initiated. During flexion, the terminal moves clockwise towards the initial and results in a negative number. Abbreviation: S, sacrum; T, thoracic spine; HROM, hip range of motion.

A total of sixteen markers were attached to the skin (right side) to the pre-palpated anatomical landmarks, marked with a pencil by an experienced physiotherapist and four markers were attached to the custom-made station Figure 1. Three custom-made spinal-clusters were positioned at the sacrum, 9^th^ thoracic spinous process, and 4^th^ thoracic spinous process. All markers were fixated to the skin by double-sided adhesive tape. All cluster-markers were additionally supported by an elastic band (Fabrifoam^®^) and Fixomull®strech tape (BSN Medical). The three-dimensional positions of the markers were determined with an accuracy of up to 0.1 mm and resolution of 0.01 mm utilizing three Optotrak^®^ cameras (Northern Digital Inc.) (Schmidt et al., 2009) at a sampling rate of 100 Hz. The data was sampled for 15 s. The three Optotrak^®^ cameras were set in an arch and calibrated/aligned towards the station’s right-sagittal side (Supplementary Material).

## Standardized fascial diagnostic test protocol

**FIGURE 2:**
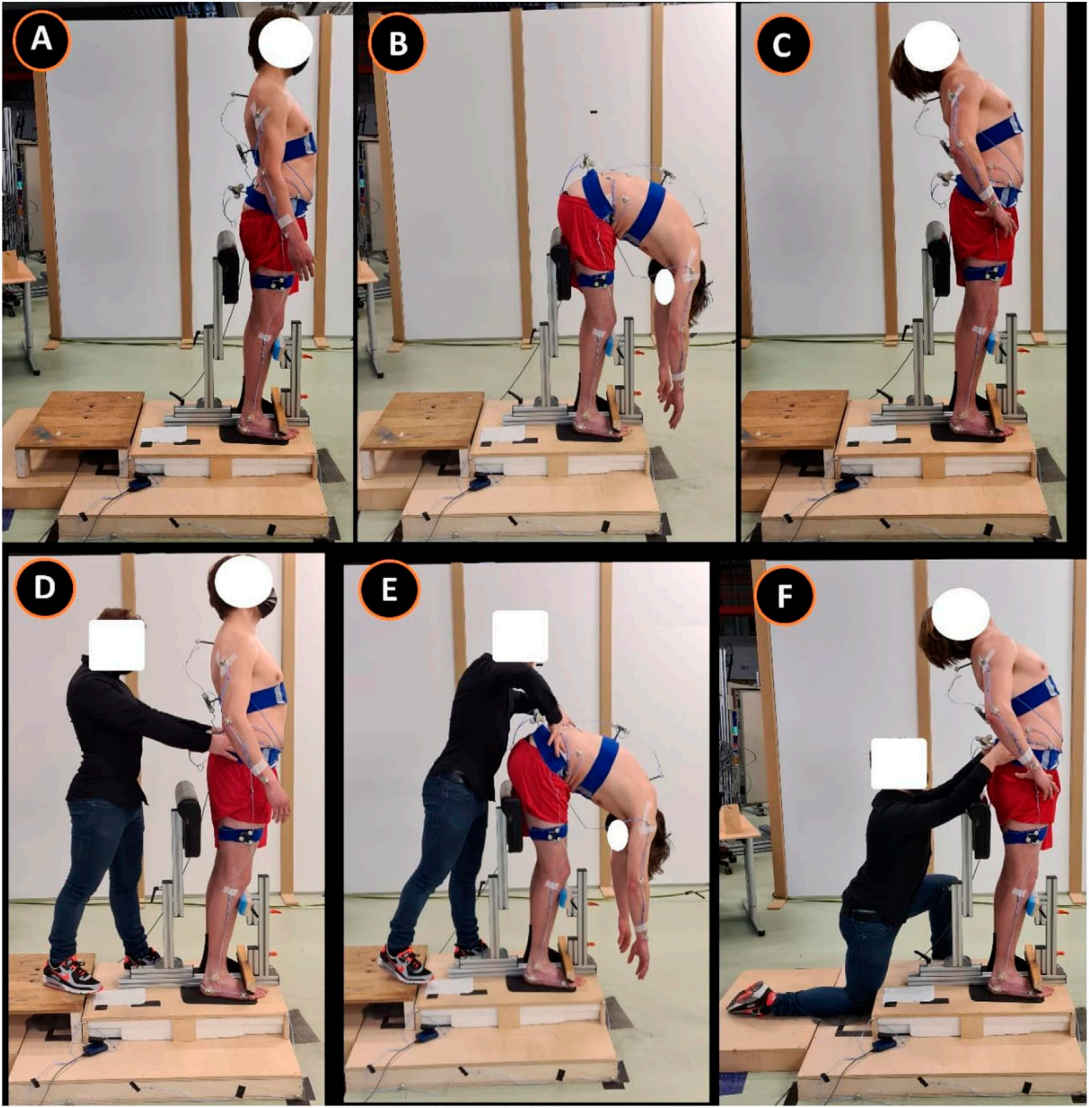
Standardized Fascial Diagnostic Test protocol. This figure represents the standardized fascial diagnostic test. Images **(A–C)** represent the spinal movement (index tests) and images **(D–F)** the spinal movements with ongoing lumbodorsal skin displacement. This research test protocol corresponds to the clinical test protocol published (Noten, 2021). **(A)**, standing neutral position; **(B)**, maximal flexion; **(C)**, maximal extension; **(D)** standing neutral position including mediolateral-directed SKD L3; **(E)**, maximal flexion including mediolateral-directed SKD L3; **(F)**, maximal extension including mediolateral-directed SKD L3. Abbreviation: SKD, skin displacement; L3, 3^rd^ lumbar spine.

**FIGURE 3:**
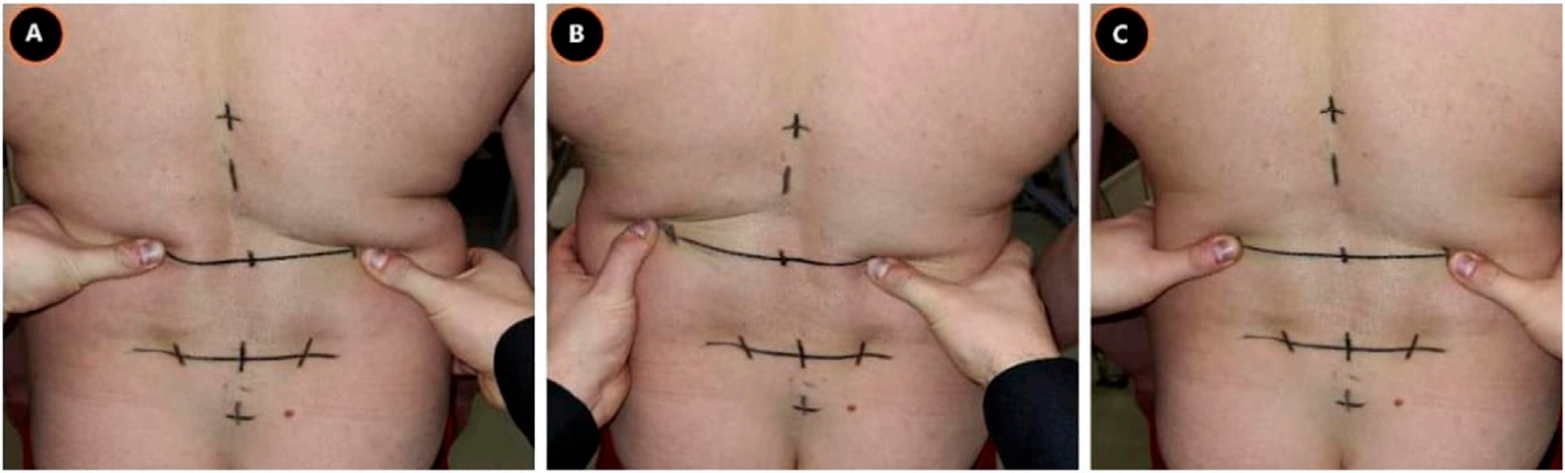
Lumbodorsal Skin-fascia Displacement. This figure represents the mediolateral-directed skin displacements at the height L3. The same displacements were performed at L5. **(A)**, Skin displacement to the right; **(B)**, Skin displacement to the left; **(C)**, Sham displacement, Abbreviation: L3, 3^rd^ lumbar spine; L5, 5^th^ lumbar spine.

Each subject started standing on a custom-made station. The custom-made station was designed in such a way that the knees could only maximally flex 10° through a leg-support. The degree of knee flexion was measured with a BASELINE®BUBBLE®INCLINOMETER. Depending on the randomization, at first, a maximal spinal flexion or extension movement (index test) was performed at own comfortable speed to retrieve the baseline reference value (repeated 3 times). In addition, either an ongoing lumbodorsal SKD or sham displacement was carried out, conform the four conditions (combination: location and direction) of the test procedure (Figure 2). The four conditions consisted of: 1) a mediolateral directed SKD to the Right (R) or Left (L) direction with respect to the spine (Figure 3) at 2) the location L5 or L3 (RL5, LL5, RL3, LL3). The SKD intensity was beyond the skin and underlying fascia slack (grade 4) equivalent to Maitland’s passive tissue stretch grading scale (Lee, 2001; Chester et al., 2003). For sham, the hands were placed with a light touch at the same locations (L5 and L3) without movement of the skin. Per condition each end-flexion or extension position (attained at the end of the movement) had to be held for 4 s (400 frames). An *a priori* experiment demonstrated no carry-over effects of the index tests when a 30-s pause was held between every single test. All above-described tests were repeated three times (1^st^, 2^nd^, and 3^rd^ test). The whole procedure was repeated twice with ongoing SKD applied by two separate testers (both experienced physiotherapists) to determine the SKD reliability through the agreement between two testers (1^st^ vs. 2^nd^) and a third time by tester 1 to determine the within tester consistency (1^st^ vs. 3^rd^), see Supplementary Material.

### Data analysis

The flexion- and extension range of motion (ROM) were assessed to estimate the mobility. The spine, pelvis, and hip complex were divided into 3 regions to analyze the thoracic, lumbar, and hip ROM changes (resp. TROM, LROM, and HROM) during flexion and extension. In addition, the distance between the wrist marker and 1st station marker was measured as the Finger Floor distance (FFD) (Gauvin et al., 1990). To calculate the angles between all 3 regions and the FFD during all conditions, two-dimensional coordinates within an XZY- Cartesian plane were used.

ROM changes were studied by determining the theta rotation (θ) inverse tangent (tan^−1^). Before the data analyses started the raw data (C3D files) was displayed in Mokka^©^, a motion kinetic and kinematic analyzer, for evaluating the calibration process and marker acquisition (Barre and Armand, 2014). The Optotrak^®^ data (NDF files) was used for data analysis utilizing a custom-made MATLAB script. Kinematic noise was filtered using a Butterworth Filter 4th order dual-pass with a cut-off frequency of 2.0 Hz. Ultimately, data was transformed into four-quadrant degrees ( rad/π*180 ), and FFD was expressed in cm.

**FIGURE 4:**
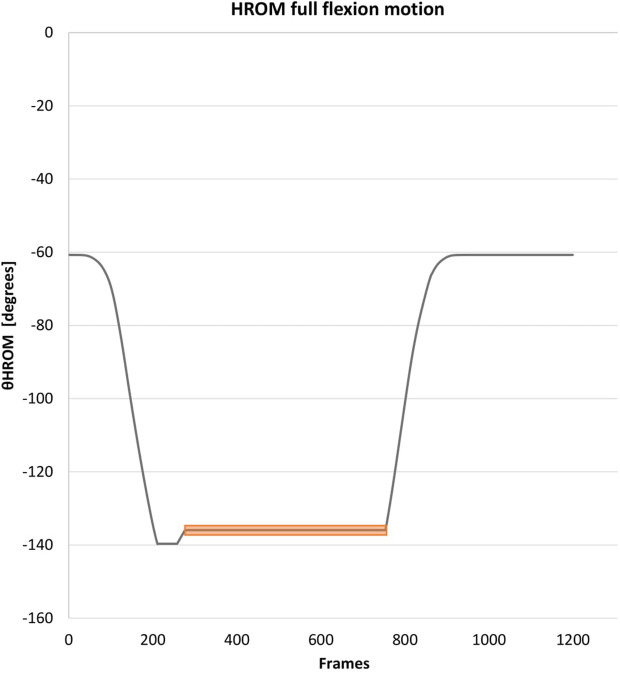
Data collection of the range of motion: Example HROM. This figure represents the data collection at the end of the hip movement. The same method was utilized for the other regions. The orange bar represents the used time frame. The peak (often found at the beginning of the time frame in end position) was corrected in the data. Abbreviation: HROM, hip range of motion.

For representation of the thoracic, lumbar, and hip region, all angles (θ) were determined by creating a tangent line from the cranial marker (C) to the medial marker (A), connecting medial marker (A) with the caudal marker (B) and creating a CAB vertex (∠CAB) (Figure 1). The CA vector was set as the terminal and the AB vector as the initial. When the terminal and initial vectors were collinear it was defined as an angle of 180°. The thoracic θ was determined by connecting the markers: T4-thoracolumbar centroid-femur centroid, lumbar θ by creating a ∠CAB by connecting markers: thoracolumbar centroid, sacrum centroid, and femur centroid, and hip θ by creating a ∠CAB by connecting markers: ilium, sacrum centroid, and femur centroid (Figure 1). The per-region flexion–and extension ROM average was calculated utilizing minimal 300 frames of the 400 frames in the end position of the index test to diminish the influence of movement towards and from this end position and allow muscle relaxation Figure 4.

To prepare the data for statistical analysis, the mean values were calculated for the three baseline tests and the three SKD tests. Furthermore, mean changes were calculated between TROM, LROM, HROM, and FFD concerning the mean baseline test per group and condition. To study the effect of SKD *versus* sham on the thoracic, lumbar, and hip ROM (aim a), the interval data was transformed into absolute data (change or no change).

### Statistical analysis

Statistical analysis was performed using SPSS^®^ (version 27.0). An outlier labeling rule based on Interquartile Range with a 2.2 multiplier (Hoaglin et al., 1986) was used in conjunction with boxplots to detect outliers. Extreme outliers in both row and/or column were excluded. Both datasets were tested for normality with the Kolmogorov Smirnov test. The absolute data was not normally distributed (kurtosis) and had to be transformed with the square root method ( SQRT X+0.5 ) (Yamamura, 1999). An *a priori* carry-over effect analysis was performed for order of testing utilizing One-way repeated ANOVA and X^2^.

A mixed-model ANOVA was used, to study the effects of SKD on thoracic, lumbar, and hip ROM *versus* sham, with 1 between-group variables (SKD group vs. sham group) and 4 within-group variables (SKD: RL5, LL5, RL3, LL3), at first using the absolute data (change or no change). Before the mixed-model ANOVA Mauchly’s test of sphericity was evaluated; an epsilon adjustment (Greenhouse Keiser at ε < 0.75, Huynh-Feldt at ε > 0.75) was used due to a lack of sphericity. A Bonferroni post HOC test was used to evaluate the difference between SKD conditions per region. The magnitudes of the effect size for all conditions were calculated with the partial eta squared ( $\eta_{p}^{2}$ ). A $\eta_{p}^{2}$ between 0.01 and 0.06 was considered as a low, a $\eta_{p}^{2}$ between 0.06 and 0.014 as a moderate, and above 0.14 as a large effect (Pierce et al., 2004; Maher et al., 2013; Rafieyan et al., 2014). Interval data was used to test if an increase or decrease in ROM occurred due to SKD per condition. *A priori* the Minimal Detectable Change (MDC_95%_) $\left(1.96*{SEM}_{consistency}*\sqrt{2}\right)$ was analyzed with the cross-tabulation z-test since ROM change should surpass the MDC_95%_.

The SKD reliability was determined by the intraclass correlation coefficient (ICC). Model 3,k for intertester reliability (ICC_3,k_) was calculated and model 3,1 for intratester reliability (ICC_3,1_) was calculated. An ICC below 0.5 was considered poor, an ICC between 0.5 and 0.75 as moderate, between 0.75 and 0.9 as good, and above 0.9 as excellent (Koo and Li, 2016). The level of significance was 0.05 for all statistical tests.

## Results

**FIGURE 5:**
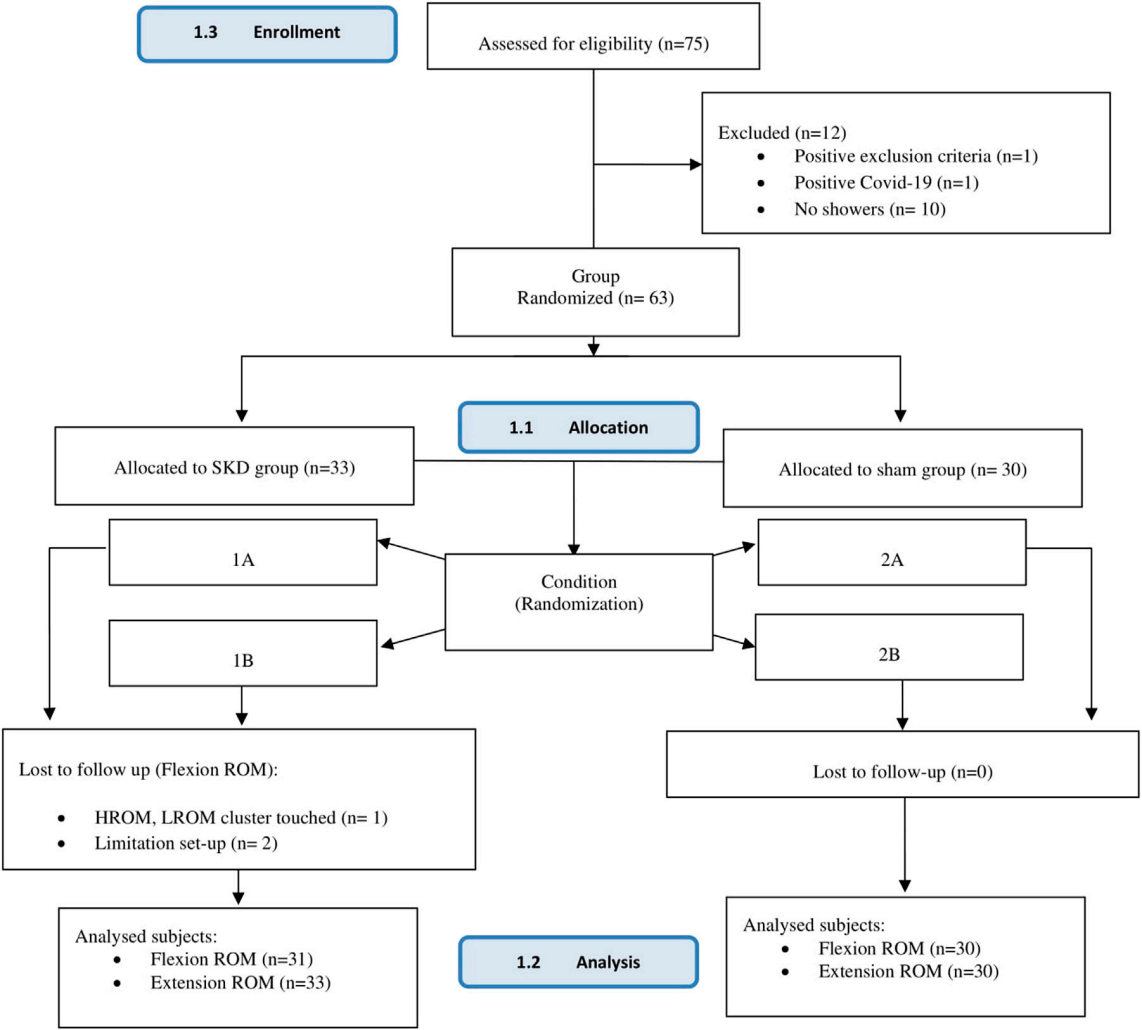
CONSORT diagram of the study.

Total seventy-five subjects were registered for the study. Twelve subjects were excluded for several reasons Figure 5. Sixty-three subjects, 26 women and 37 men, age 35 ± SD1.18 years were enrolled for the study. No significant differences in demographic characteristics between groups (SKD *n* = 33; Sham *n* = 30) were present Table 1.

**TABLE 1:** Demographical statistics. This table describes the participant characteristics: Mean (±SD) age, length, body weight, body mass index. *p*-values from an independent samples *t*-test between the SKD group and sham group are shown within each group. No significant differences presented (*p* > 0.05). Abbreviations: SD, Standard deviation; P, Significant differences (*P* < 0.05) between groups.

Variables	Total	Range	SKD group (*n* = 33)	Sham group (*n* = 30)	*P*
	Mean (SD)		Mean (SD)	Mean (SD)	
Age (Years)	35 (1.18)	25–54	35 (9)	34 (9)	0.769
Length (centimeters)	176 (0.10)	156–194	177 (0.10)	174 (0.09)	0.053
Weight (kilogram)	73.46 (1.27)	51.20–92.50	76 (9.5)	71 (10.2)	0.118
Body Mass Index	23.69 (0.33)	18.36–30	24.10 (2.70)	23.24 (2.40)	0.192

For one subject the HROM and LROM data points were excluded since the sacrum cluster was touched during the flexion measurements. In addition, for two subjects the flexion data were excluded due to exceeding the large but still limited range of motion in the experimental set-up. The processed data consisted of a total of 3276 records (flexion, *n* = 1872; extension, *n* = 1,404). Thirty flexion and 46 extension data points were detected as extreme outliers and were excluded from analyses. The variables within each group had a normal distribution (*p* < 0.05). No significant carry-over effects were found regarding the order of testing (*p* < 0.05). The square root transformed data was back-transformed for interpretation.

### SKD effect on thoracic, lumbar, and hip range of motion

**FIGURE 6:**
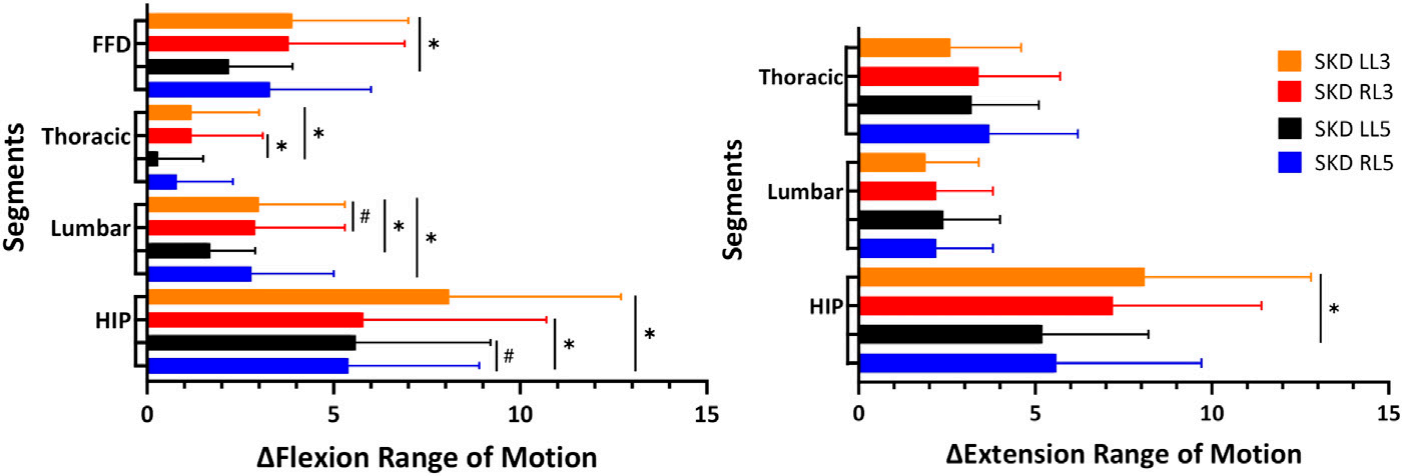
Thoracic, lumbar, and hip mobility difference between SKD conditions.This figure represents the absolute mean difference between the Skin Displacement (SKD) conditions on the Range of Motion. The x-axis represents the Range of Motion in degrees for regions and centimeters for FFD. The black lines represent the standard deviation of 95% with “✱” the significant difference found between location (*p* < 0.05) and “#” the significant difference found between direction (*p* < 0.05). Abbreviation: SKD, skin displacement; L3, 3^rd^ lumbar spine; L5, 5^th^ lumbar spine; RL5, Right SKD at L5; LL5, Left SKD at L5; RL3, Right SKD at L3; LL3, Left SKD at L5.

Differences between SKD conditions per region are displayed in Figure 6. The assumption of sphericity for flexion ROM within the SKD group was violated for HROM (Mauchly’s W = 0.759, *p* < 0.007, ε > 0.75), not for LROM, TROM, and FFD (*p* > 0.05). No group and condition interaction effects were found for all regions (*p* < 0.05). For all regions, the mean flexion ROM was significantly greater in the SKD group (*p* < 0.05) Table 2. Post hoc testing revealed that only SKD affected flexion ROM (for all regions) which was dependent on the SKD condition used (*p* < 0.001). Detailed information regarding the SKD data is presented in Table 3.

**TABLE 2:** Thoracic, lumbar, and hip baseline-post change: between groups difference. The results presented are the absolute results of baseline minus the experimental SKD utilized in the SKD group and the absolute results of baseline minus sham SKD utilized in the sham group. Significant differences (*p* < 0.05) between the groups are represented. Abbreviations: F, Flexion; E, Extension; H, Hip; L, Lumbar; T, Thoracic; ROM, Range of Motion; FFD, Finger Floor Distance; SD, Standard deviation; P, Significant differences (*P* < 0.05) between groups.

Index test	Variable	SKD group		Sham group		F value	*P*	Effect size
		Mean (SD)		Mean (SD)		η^2^ *_p_*		
	FHROM	6.0	(2.6)	2.6	(1.8)	34.238	0.001	0.86
Flexion	FLROM	2.7	(1.3)	1.6	(1.1)	12.919	0.001	0.90
	FTROM	1.1	(0.7)	0.5	(0.5)	15.381	0.001	0.12
	FFD	2.6	(1.8)	1.5	(1.4)	8.571	0.005	0.50
	EHROM	6.3	(2.3)	2.6	(1.6)	53.599	0.001	0.42
Extension	ELROM	2.4	(1.0)	1.6	(0.9)	9.828	0.003	0.29
	ETROM	3.4	(1.5)	2.5	(1.3)	6.542	0.013	0.38

**TABLE 3:** Mediolateral SKD comparison. The results presented are the absolute results of baseline minus the experimental SKD utilized in the SKD group and the absolute results of baseline minus sham SKD utilized in the sham group. Significant differences (*p* < 0.05) between the SKD conditions are represented. Abbreviations: F, Flexion; E, Extension; H, Hip; L, Lumbar; T, Thoracic; ROM, Range of Motion; FFD, Finger Floor Distance; SD, Standard deviation; P, Significant differences (*P* < 0.05) between SKD conditions.

Variable	Within conditions								Within factor		Effect size	Pairwise comparison		
	1.RL5		2.LL5		3.RL3		4.LL3							
	Mean	(SD)	Mean	(SD)	Mean	(SD)	Mean	(SD)	F value	*p*-value	η^2_*p*_^	Condition	Pairs	P
FHROM	5.4	(3.5)	5.6	(3.6)	5.8	(4.9)	8.1	(4.6)	7.334	0.001	0.14	LL3-RL5	location	0.021
												LL3-LL5	location	0.026
												LL3-RL3	direction	0.002
EHROM	5.6	(4.1)	5.2	(3.0)	7.2	(4.2)	8.1	(4.7)	3.732	0.022	0.13	LL5-LL3	location	0.016
FLROM	2.8	(2.2)	1.7	(1.2)	2.9	(2.4)	3.0	(2.3)	4.636	0.009	0.12	LL5-RL3	location	0.038
												LL5-LL3	location	0.027
												LL5-RL5	direction	0.041
FTROM	0.8	(1.5)	0.3	(1.2)	1.2	(1.9)	1.2	(1.8)	4.997	0.006	0.17	LL5-RL3	location	0.009
												LL5-LL3	location	0.009
FFD	3.3	(2.7)	2.2	(1.7)	3.8	(3.1)	3.9	(3.1)	3.809	0.020	0.92	LL5-LL3	location	0.033

The assumption of sphericity for extension ROM within the SKD group was violated for all regions (*p* > 0.05, ε > 0.75), hence, Huynh-Feldt correction was used for data interpretation. For all regions, the extension ROM was significantly greater in the SKD group (Table 2). No group and condition interaction effects were found for LROM and TROM (*p* < 0.05). SKD only affected LROM and TROM which was not different between SKD conditions. An interaction effect of group and condition was found for HROM (F = 2.646, *p* = 0.55). Within the SKD group, the affected HROM was dependent on the SKD condition applied (F = 3.732, *p* = 0.022). Post hoc analysis revealed that the HROM was differently affected between SKD conditions LL5 and LL3 (*p* < 0.05) (Table 3). No significant effect was present in the sham condition. However, the affected LROM and TROM did not differ between the SKD conditions. The effect size in change of flexion- and extension ROM by SKD was large (Effect size: flexion $\eta_{p}^{2}$ = 0.12–0.90; extension $\eta_{p}^{2}$ = 0.29–0.42).

To demonstrate a significant effect on flexion ROM, the change should be larger than the MDC_95%_ values (Table 4), which were for HROM (MDC_95%_ = 2.9), LROM (MDC_95%_ = 1.5), and TROM (MDC_95%_ = 0.9). In most SKD conditions a decrease in flexion ROM occurred for all regions, except for the thoracic which increased during SKD LL3, (*p* < 0.05) (Table 5).

**TABLE 4:** Minimal Detectable change of the SKD on mobility. The Standard Error of Measurement and Minimal Detectable change 95% are calculated utilizing Model 3,k for intertester reliability. Abbreviations: F, Flexion; E, Extension; H, Hip; L, Lumbar; T, Thoracic; ROM, Range of Motion; FFD, Finger Floor Distance; Δ, baseline-SKD difference; ICC, consistency intraclass correlation coefficient; CI, 95% confidence interval; SEM, Standard Error of Measurement, MDC, Minimal Detectable Change; X, doesn’t exist.


Region	Δ HROM	Δ LROM	Δ TROM	Δ FFD
	ICC_3,k_ (95% CI) SEM	ICC_3,k_ (95% CI) SEM	ICC_3,k_ (95% CI) SEM	ICC_3,k_ (95% CI) SEM
	MDC_95_	MDC_95_	MDC_95_	MDC_95_
Flexion	0.82 (0.72–0.88)	0.82 (0.72–0.87)	0.78 (0.67–0.85)	0.81 (0.70–0.87)
	1.0	0.6	0.3	0.8
	2.9	1.5	0.9	2.1
Extension	0.93 (0.90–0.96)	0.78 (0.67–0.85)	0.84 (0.72–0.85)	X
	0.6	0.5	0.6	
	1.7	1.4	1.6	

**TABLE 5:** Range of Motion increased and decreased. This table describes the significant difference between groups and counts/percentage of subjects who achieved the Minimal Detectable change 95% per region based on increased and decreased range of motion. Abbreviations: F, Flexion; E, Extension; ROM, Range of Motion; HROM, Hip ROM; LROM, Lumbar ROM; TROM, Thoracic ROM; FFD, Finger Floor Distance; MDC95%, Minimal detectable change based on 95% confidence interval; MDC95%>, the change in ROM is greater than the calculated MDC; P, Significant differences (*P* < 0.05) between groups per condition.

Condition	ROM increased					ROM decreased				
	SKD group		Sham group		z-test	SKD group		Sham group		z-test
*ROM ≥ MDC*_*95%*_	*%*	*Counts*	*%*	*Counts*	*P*	*%*	*Counts*	*%*	*Counts*	*p*
FHROM RL5	55.6	5/11	44.4	4/11	NS	81.0	18/23	18.2	4/18	*p* < 0.05
FHROM LL5	—	—	—	—	—	76.7	23/23	23.3	7/7	*p* < 0.05
FHROM RL3	62.5	5/13	37.5	3/10	NS	65.0	13/21	35.0	7/19	NS
FHROM LL3	62.5	5/7	37.5	3/9	NS	71.9	23/27	28.1	9/20	*p* < 0.05
FLROM RL5	100	3/10	0	0/10	NS	83.3	15/24	16.7	3/19	*p* < 0.05
FLROM LL5	50.0	1/12	50.0	1/16	NS	83.3	10/22	16.7	2/13	NS
FLROM RL3	55.6	5/14	44.4	4/14	NS	78.6	11/20	21.4	3/15	*p* < 0.05
FLROM LL3	60.0	3/13	40.0	2/9	NS	82.4	11/21	17.6	3/20	*p* < 0.05
FTROM RL5	62.2	24/24	36.8	14/14	NS	—	—	—	—	—
FTROM LL5	76.5	13/23	23.5	4/16	NS	50.0	6/11	50.0	6/13	NS
FTROM RL3	60.0	15/20	40.0	10/16	NS	66.7	12/14	33.3	6/13	*p* < 0.05
FTROM LL3	66.7	12/13	33.3	6/12	*p* < 0.05	64.0	16/21	36.0	9/17	NS
FFD RL5	35.7	5/13	64.3	9/18	NS	80.0	16/21	20.0	4/11	*p* < 0.05
FFD LL5	57.1	4/15	42.9	3/16	NS	66.7	12/19	33.3	6/13	NS
FFD RL3	46.2	6/15	53.8	7/16	NS	66.7	14/19	33.3	7/13	NS
FFD LL3	54.5	6/13	45.5	5/11	NS	63.6	14/21	36.4	8/18	NS
EHROM RL5	69.6	16/22	7.0	7/20	*p* < 0.05	77.8	7/12	22.2	2/9	NS
EHROM LL5	87.5	21/24	12.5	3/17	*p* < 0.05	63.6	7/10	36.4	4/12	NS
EHROM RL3	77.4	24/27	22.6	7/20	*p* < 0.05	60.0	6/7	40.0	4/8	NS
EHROM LL3	81.3	26/28	18.8	6/18	*p* < 0.05	80.0	4/6	20.0	1/11	*p* < 0.05
ELROM RL5	81.3	13/28	18.8	3/19	*p* < 0.05	25.0	1/6	75.0	3/10	NS
ELROM LL5	79.2	19/27	20.8	5/16	*p* < 0.05	0.0	0/7	100	1/13	NS
ELROM RL3	85.7	12/24	14.3	2/20	*p* < 0.05	60.0	3/10	40.0	2/9	NS
ELROM LL3	84.6	11/26	15.4	2/16	*p* < 0.05	33.3	1/8	66.7	2/13	NS
ETROM RL5	72.7	16/25	27.3	6/22	*p* < 0.05	80.0	4/8	20.0	6/7	NS
ETROM LL5	70.0	14/26	30.0	6/21	NS	100	3/8	0.0	0/7	NS
ETROM RL3	50.0	7/21	50.0	7/18	NS	60.0	3/13	40.0	2/10	NS
ETROM LL3	55.8	24/24	44.2	19/19	NS	50.0	10/10	50.0	10/10	NS

Based on the MDC_95%_, extension HROM (MDC_95%_ = 1.7), LROM (MDC_95%_ = 1.4), and TROM (MDC_95%_ = 1.6) increased in almost all SKD conditions. Contrary to the foregoing, a significant extension decrease was measured for the hip during SKD LL3 (*p* < 0.05) (Table 5).

### Reliability

The intraclass correlation coefficient analysis confirmed a significant agreement in effect on thoracic, lumbar, and hip ROM between the physiotherapists who performed the SKD (*p* < 0.001) with an ICC_3,k_ for all regions (0.81–0.93). The observed affected thoracic, lumbar, and hip ROM between the repeated SKD tests performed by a physiotherapist was consistent as shown by the ICC_3,1_ ranging from 0.70 to 0.84 (Table 6).

**TABLE 6:** Intratester and intertester reliability. This table represents the intraclass correlation coefficient (*ICC*) with its 95% confidence interval (*CI95%*) for reliability. The reliability was analyzed through the ICC_3,k_ (agreement) retrieved from average measures and ICC_3,1_ (consistency) retrieved from single measures.

Region	Flexion								Extension					
	HROM		LROM		TROM		FFD		HROM		LROM		TROM	
	*ICC*	*(CI95%)*	*ICC*	*(CI95%)*	*ICC*	*(CI95%)*	*ICC*	*(CI95%)*	*ICC*	*(CI95%)*	*ICC*	*(CI95%)*	*ICC*	*(CI95%)*
ICC_3,k_	0.82	(0.72–0.88)	0.82	(0.72–0.87)	0.78	(0.67–0.85)	0.81	(0.70–0.87)	0.93	(0.90–0.96)	0.78	(0.67–0.85)	0.84	(0.75–0.89)
ICC_3,1_	0.84	(0.79 -0.89)	0.82	(0.75–0.87)	0.83	(0.77–0.88)	0.81	(0.74–0.84)	0.83	(0.76–0.87)	0.70	(0.60–0.78)	0.72	(0.62–0.79)

## Discussion

This study shows that lumbodorsal mediolateral directed SKD affects thoracic, lumbar, and hip ROM. The effects of SKD are substantially direction -and location different as well as movement (flexion/extension) specific. In general, the mean flexion ROM decreased while mean extension ROM increased. However, based on the MDC_95%_ some individuals showed an increase in flexion ROM (*n* = 23, MDC_95%_ range = HROM:2.9- 21.3, LROM:1.5–6.5; TROM:0.9–7.0) and a decrease in extension ROM (*n* = 27, MDC_95%_ range = HROM: 1.7- 9.1, LROM: 1.4- 7.0, TROM: 1.6–5.2) due to SKD which was much greater than in the sham group. To the best of our knowledge, this is the first study showing that SKD affects the thoracic, lumbar, and hip ROM based on the SKD condition used. These findings support the hypothesis that skin and underlying fasciae are modulated by SKD. Consequently, this may have an impact on the structures that determine the thoracic, lumbar, and hip ROM. Whether this effect is due to altered tensions in underlying fasciae remains to be determined.

A strength of the study is the robust internal validity of the study because of several reasons: 1) the FDT consisted of a standardized procedure that was performed in a custom-made station in reducing the change of random errors (Gauvin et al., 1990), 2) the wrist instead of the finger-tip-floor distance was utilized to register FFD (Akaha et al., 2008), 3) SKD was performed by two trained physiotherapists, 4) sample size was sufficient (*n* = 63), 5) the order of SKD conditions per group was randomized, and 6) the increase or decrease of ROM per region was based on surpassing the MDC_95%_.

A limitation could have been that the sham skin displacement chosen may have differed from the SKD conditions not only in the lack of horizontal shear stress (mediolateral displacement) but also in the much lower normal stress (posterior-anterior pressure) used in the sham group. Previous studies have shown that the amount of pressure experienced by patients may influence the treatment effects (Wilson et al., 2021) and also that posterior-anterior pressure on the skin without any mediolateral displacement influences joint mobility (Cagnie et al., 2013; Takamoto et al., 2015). It cannot be completely ruled out, that the observed mobility changes in the SKD group in this study were not only caused by the discussed effects of a mediolateral displacement but by the differences in the experienced pressure by the subjects in the SKD group *versus* the sham group. Another limitation could have been that the subjects were unfamiliar with performing the index tests in a fixed position on an elevated station (20.5 cm), which could have increased fear. Fear is associated with increasing myoelectric activity of the lumbar erector muscles in healthy subjects (Pool-Goudzwaard et al., 2018) influencing the thoracic, lumbar, and hip ROM (Colloca and Hinrichs, 2005). This might have affected the observed ROM. To diminish a possible activation of muscles, subjects were asked to stay in the end position for 4 s to stimulate muscle relaxation (McGorry and Lin, 2012). The thoracolumbar cluster T12-T8 that was used in our study was not fully in line with the more commonly used T12-L1 location (Vazirian et al., 2016). We have chosen this cluster marker since a pilot experiment showed that the markers of T12 could not be detected during the flexion- and extension movements. Finally, the second measurement was always performed by a different physiotherapist. Should an effect have occurred between measurements, the differences in thoracic, lumbar, and hip ROM outcome measures between the 1^st^ and 3^rd^ index tests would have been greater than the differences in measures between the 1^st^ and 2^nd^ index tests because repetition of movements increases ROM (Holzgreve et al., 2020). No carry-over effects were demonstrated, indicating no increase in thoracic, lumbar, and hip ROM during the 1^st^, 2^nd^, and 3^rd^ tests.

## Implications

This study is the first step in investigating the Dynamic ArthroMyofascial Translation^®^ Test by evaluating the SKD effects on the spine, pelvis, and hip ROM (Noten, 2021). The next step will be to test whether SKD affects the spine, pelvis, and hip ROM in low back pain subjects and to test the implications for clinical application.

The initial SKD intensity aim was grade 4 (Lee, 2001; Chester et al., 2003). The question is whether both testers applied the SKD at a similar intensity. Since the intertester- and intratester reliability was good (resp. ICC_3,k_ = 0.81–0.93; ICC_3,1_ = 0.70–0.84) it is likely that amplitude, displacement, and force applied to the skin were fairly similar for the two experienced physiotherapists. It remains to be determined whether a difference in intensity between less and more experienced physiotherapists exists. The SKD is increasingly used by physiotherapists during physical examination to determine whether or not a patient would benefit from FTMs. However, this needs to be assessed. It is conceivable that for clinical purposes fascial diagnostic testing needs to be adapted and optimized. Moreover, whether the same effects of SKD occur in people with low back pain needs to be investigated.

### The underlying mechanisms of skin displacement?

The rationale underlying the SKD effects is that the skin is an important structure that allows force transmission onto underlying structures. The generated (normal- tensile- and shear) force during SKD is expected to be transmitted *via* the lumbodorsal superficial fascia to the thoracolumbar fascia, myofascia, muscles, and thoracic, lumbar, and hip arthrofascia since they are linked *via* connective tissues (Willard et al., 2012; Herlin et al., 2015; Adamietz et al., 2021). These forces, strain the aforementioned structures and may influence the interfascial and fasciae-muscle relative positions (Huijing and Baan, 2003). This could affect the stiffness in fasciae and skeletal muscles (Huijing and Baan, 2003; Maas, 2019; Ruttiman et al., 2019) which is modulated by (activating or deactivating) mechanoreceptors (proprioceptors, kinesthetic-receptors, some nociceptors). This might lead to altered skeletal muscle contractions, thereby, affecting the spine, pelvis, and hip ROM (Huijing and Baan, 2003; Maas, 2019).

In support of this rationale, most SKD conditions did not increase the flexion ROM and did not decrease the extension ROM. Subsequently, the affected flexion- and extension ROM differs between the SKD conditions. We hypothesize that changes occurs in: 1) fasciae position, 2) stiffness, and/or 3) agonist- and antagonistic muscle activity. Understanding the underlying mechanisms requires a different experimental design with other measurement instruments like ultrasonography and electromyography.

## Conclusion

This study shows that lumbodorsal SKD affects the spine, pelvis, and hip range of motion in healthy subjects. Flexion ROM decreased by SKD whereas extension ROM increased. The range of motion change depended on the SKD location and direction. The reliability of the SKD was remarkably good. These results suggest that the SKD may be a promising interventional test to obtain an indication, whether or not, a patient would benefit from FTMs. Further research is warranted to obtain insight into the mechanisms by which SKD affects the spine, pelvis, and hip range of motion, muscle activation, force transmission, in healthy, asymptomatic, and low back pain subjects.

## Data Availability

The raw data supporting the conclusion of this article will be made available by the authors, without undue reservation.

## Glossary

### Mobility

F: flexion
E: extension
ROM: range of motion
HROM: hip ROM
LROM: lumbar ROM
TROM: thoracic ROM
FFD: finger floor distance

### Skin Displacement

FTMs: fascia tissue manipulations
FTM: fascia tissue manipulation
SKD: skin displacement (mediolateral)
L5: 5^th^ lumbar segement
L3: 3^rd^ lumbar segment
RL5: Right SKD at the height L5
LL5: Left SKD at the height L5
RL3: Right SKD at the height L3
LL3: Left SKD at the height L3

### Statistics

SD: Standard deviation
Δ: the difference between the index motion test and motion with ongoing SKD on the range of motion
ICC: intraclass correlation coefficient
CI95%: the 95% confidence interval level
SEM: standard error of measurement
MDC: minimal detectable change
NS: not significant
ABS: absolute value
MDC_95%_: minimal detectable change based on 95% confidence interval
