# Supplementary material for: Skin Displacement as fascia tissue manipulation at the lower back affects instantaneously the flexion-and extension spine, pelvis, and hip range of motion
Source: Front Physiol. 2022 Nov 23;13:1067816. doi: 10.3389/fphys.2022.1067816 (PMC9727291; doi:10.3389/fphys.2022.1067816)
Supplement: Supplementary file 1 [file DataSheet1.pdf]

Supplement Research Protocol

(VCWE-2020-095R1)

Skin Displacement as Fascia Tissue Manipulation at the lower back affects the flexion- and extension spine, pelvis, and hip range of motion

Robbert N. van Amstel, MSc

Prof. Dr. Richard T. Jaspers

Prof. Dr. Annelies L. Pool-Goudzwaard

Faculty of Behavioural and Movement Sciences, Vrije Universiteit Amsterdam, Amsterdam Movement Sciences, The Netherlands

ORCID:

van Amstel: https://orcid.org/0000-0002-4641-0094

Jaspers: https://orcid.org/0000-0002-6951-0952

Pool-Goudzwaard: https://orcid.org/0000-0001-8524-2516

Introduction

Regarding the effectiveness of Fascia Tissue Manipulations (FTMs) on pain and mobility, it has been proposed that this will depend on both location and direction of the applied skin displacement (SKD) (Noten, 2021). It has been proposed that SKD will affect fasciae stiffness and their relative positions to surrounding tissues (Huijing and Baan, 2003; Maas, 2019), which can be beneficial but may also 'worsen' pain and decrease mobility (Noten, 2021). To indicate whether or not a patient would benefit from FTMs, a fascial diagnostic test has been proposed: The Dynamic ArthroMyofascial Translation® Test (OSF, https://osf.io/52ze7). The test consists of 3 steps: 1) affirmation of the most painful movement from stance to either flexion or extension, as a reference test, 2) the same test with ongoing mediolateral directed SKD to the right at e.g. L3 or L5, and 3) the same reference test with on-going SKD to the left. SKD leading to the largest mobility improvement and/or pain reduction can be utilized for FTMs at the tested location (Noten, 2021).

Several studies in which FTMs have been applied to healthy humans by elastic tape or myofascial release have shown that fasciae and muscles below the skin undergo deformations and are locally strained (Tu et al., 2016; Wong et al., 2017; de las Penas, 2019; Wang et al., 2019). Therefore, it is conceivable that variable effects in alterations in mobility (i.e., increase or decrease) due to FTMs by SKD are also expected to occur in healthy subjects, but could be less pronounced than in patients with limited mobility for instance in case of low back pain.

Although changes in joint mobility through SKD seem to be clinically effective, the basal effects of SKD on healthy subjects have not been tested objectively. Therefore, this research test protocol has been developed corresponding to the clinical test protocol published (Noten, 2021). The aims of this study were: a) to assess whether SKD at the lower back affects flexion- and extension range of motion of the spine, pelvis, and hip complex versus a sham skin-displacement, in healthy subjects, and b) if present, to test the effect of SKD at different locations and directions, as well as c) to assess intertester and intratester reliability of applying the SKD.

Contents

1. Preparation of the experimental setup 4
   1.1. Requirement list: 4
   1.2. Marker, strober & CSU setting.. 4
   1.3. Motion capture position 4
   1.4. Preparation and marker placements 5
2. Carry-over effects 6
3. Standardized Fascial Diagnostic Test protocol 6
   3.1. Execution index test and division tasks 6
   3.2. Pre-instruction physical therapist.. 7
   3.3. Index tests.. 7
   3.3. Experimental- and SHAM Skin Displacement Maneuver.. 8
4. Skin Displacement condition orders 9
5. Legends to the figures 10
6. MATLAB script...29

1. Preparation of the experimental setup

1.1. Requirement list:

1) 3 x Optotrak® cameras (Northern Digital Inc.)
2) 1 x mobile control unite
3) 1 x cube 16 rigid body (calibration)
4) 1 x optototrak cube adapter
5) 16 x retro-reflective markers

1.2. Marker, strober & CSU setting

In total, two strobers (1 for the subject and 1 for the custom-made station) were utilized. The connection process is depicted in **Figure 1**.

In maintaining consistency for data processing, during every measurement, the markers were linked in the same order, also, the marker-connectors were plugged into the same strober portals, and both strobers into the same system control unit (SCU) portals.

The strober marker-linkages order is described in order from cranial to caudal marker:

<u>Subject 1st strober marker-linkages:</u>

Strober portal 1: 4th thoracic spinal process (T4), thoracolumbar junction, sacrum (cluster markers)

Strober portal 2: Wrist, elbow, shoulder

Strober Portal 3: MTP 5, calcaneus, malleolus lateraal, femur cluster (n=3), medial border crista

Strober Portal 3: Sacroiliac anterior superior joint (SIAS), sacroiliac posterior superior joint (SIPS)

- Sixteen markers in 1st strober which is connected in CSU portal 1.

<u>Station 2nd strober marker-linkages:</u>

Strober Portal 1: one each station angle a marker was adherend (n=4)

- Four markers in 2nd strobes which are connected in CSU portal 2.

1.3. Motion capture position

The three Optotrak® cameras were set and aligned within an arch. The custom-made station setup was counterclockwise so that the right side of the custom-made station was perpendicular to the Optotrak® camera -1 y-axis (sagittal plane). The other two cameras (2 & 3) were placed at an angle of

+ - 35° degrees towards the custom-made station. The distance between the cameras and the lateral border of the station was 4 meters for optimal visualization. The three Optotrak® cameras were connected via a linking cable, of which the connector of the last cable was placed in the CSU **(Figure 2)**.

1.4. Preparation and marker placements

A total of sixteen active infrared markers were attached to the skin to the pre-palpated anatomical landmarks, marked with a pencil by a 10 year experienced physical therapist (right side) and four markers were attached to the custom-made station (right side):

1) Six markers represented the right leg:
- Metatarsofalangeaal, os calcaneus, lateral malleolus, femur cluster (3 marker-cluster)
2) Six markers represented the spinal column:
- Sacrum (3 marker-cluster), thoracolumbar junction (T8-T12: 2 marker-cluster), 4th thoracic spinal process (T4)
3) Three markers represented the right arm:
- Tuber deltoid, os olecrani, processus styloid radii
4) Other three:
- Medial Crista illicus, SIPS, SIAS

Three custom-made spinal-clusters were positioned at the sacrum, 9th thoracic spinous process, and 4th thoracic spinous process. All markers were fixated to the skin by double-sided adhesive tape. All cluster-markers were extra supported by an elastic band (Fabrifoam®) and Fixomull®strech tape (BSN Medical) (Figure 5A).

2. Carry-over effects

In analyzing the SKD effect on the spinopelvic-hip mobility various actions were taken to minimalize measurement errors. To wash out the warming-up effect of the repeated index test and the SKD carryover effects, a rest period of 30 seconds was inserted between each single performed mobility test. As well, the applied SKD condition (location incl. location) was mixed in order in different sequences. Besides, the 1st index test was followed by the opposite 2nd index test (1st flexion followed with 2ndextension or 1st extension followed with 2nd flexion). Each 1st and 2nd index test with its 2 corresponding conditions was performed 3 times within a cluster (n= 6). After a cluster was completed, a rest period of 180 seconds was inserted in washing out warming-up effects before performing the 2nd cluster. Between the rest periods 30, 120, and 180 seconds the subject remained stationary. After performing the first SKD condition-order (e.g. 1A) a ten minutes break had been inserted before the subject crossed over to the other equivalent SKD condition-order (e.g. 2A)**.** In these ten minutes, the subject was disconnected from the Optotrak® system and was allowed to walk around the lab but was not allowed to sit down **(see flowchart, Figure 3)**.

3. Standardized Fascial Diagnostic Test protocol

3.1. Execution index test and division tasks

Two researchers performed and directed the standardized procedure, in minimalizing and controlling the external errors **(Figure 4)**.

Task Physical therapist_1 or 2: The physical therapist instructed the subject_d how the index tests should be performed.

Task Researcher_a: The researcher who monitors the computer also gave the cues, because he was the only one who could overview the registration system (time of recording).

Task Researcher_b: Before the test started, researcher_b showed the information given on the block card **(Figure 9)** to the physical therapist concealed from the subject. Besides, he/she monitored the subject's end of the spinopelvic-hip ROM and controlled that the subject stayed in this position for four seconds, monitored with a stopwatch. See for index test instruction **§3.3 and table 1**.

3.2. **Pre-instruction physical therapist**

A detailed explanation was discussed with the subject before the execution of the tests. After the markers were applied, the subject was instructed how the index test should be performed. Physical therapist_2 took place on the station where physical therapist_1 briefly explained how the test should be performed. During this explanation, physical therapist_2 executed the instruction for visualization for subject clarity. After this, the subject was asked to stand on the station platform and was hooked to the CSU. The station was set in such a way that the knee could only flex till 10 degrees from the neutral standing position to end trunk flexion. This was controlled with a BASELINE®BUBBLE®INCLINOMETER. When everything was set and the subject was familiar with the index tests, the extended instruction was performed where each index test was practiced 10 times.

After the baseline, before the tests started, the following sentence was communicated to the subject (SKD- and sham-group) by the physical therapist, *" you may perform the index test as we have practiced before, one of us will stand behind you and palpate some points on your back, 'you do not have to do anything with this then only performing the index test at the given cue"*.

3.3. **Index tests**

For this purpose, the spinopelvic-hip flexion- and extension motion was used as an index test in evaluating the SKD effect on spinopelvic-hip ROM, performed as follow:

1. <u>Start position:</u> the subject stood straight up, gaze forward, with arms hung along its thighs, thumbs pointed forward, and feet stood parallel and contiguous **(Picture 5A)**.
2. <u>Flexion:</u> At the cue for flexion, the subject brought his chin to his chest and rolled it down from torso to lower back, and bent as far as possible without forcing. During this flexion movement, the subject's shins were placed against the shin support and the hamstrings towards the leg support in securing the 10 degrees flexion. At the end ROM the subject relaxed and hung out for four seconds **(Picture 5B)** and came back to starting position.
3. <u>Extension:</u> From starting position on the cue for trunk extension the subject placed its hands on his trochanter major. Subsequently, the subject extended its head followed by extending its torso to the lower back till the end range was reached. This position was held for four seconds without forcing the extension **(Picture 5C)**. During this extension movement, the subject shins were placed against the shin support without contacting the hamstrings with the leg support.

3.3. Experimental- and SHAM Skin Displacement Maneuver

From starting position (standing tall) the subject its skin and underlying fasciae were manually lateral-horizontal displaced by the physical therapist utilizing the SKD. During the starting position, the subject was stationary, and the physical therapist stood behind the subject.

Researcher_a monitored the rest periods (timing turning on the optotrak®system). Researcher_a called out,''10 seconds to go'', which informed the physical therapist to palpate the reference point **(Picture 6A)** displayed on the block card **(Figure 9)**. Ten seconds later researcher_a called out ''camera set'' which was the cue for the physical therapist to apply the SKD **(Picture 6B or 6C)** in de direction and location displayed on the block card which was followed with the cue,'' GO'', which triggered the subject to perform the index test. During this movement, the physical therapist performed the SKD and held the skin and underlying fasciae under tension till the subject was back in the start position **(Picture 7A and 8A)**.

For example: the physical therapist simultaneously placed his left thumb on the left reference point and his right thumb at the right reference point at the height of the spinous processes (e.g. L3) **(Picture 6A)**. From this point, the physical therapist executed the SKD by shifting the skin horizontally by pushing and pulling and holding the skin and underlying fascia under tension with optimal force, enough to take up the skin/fascia slack (grade 4 Maitland) in a certain direction (condition dependent) **(Picture 6B & 6C)** during the subject's full spinopelvic-hip motion **(Picture 7B & Picture 8B)**. The same procedure was performed in the sham-group with a light touch at the same locations (L3 and L5) without movement of the skin. When the researcher_a called out, ''10 seconds to go'', as well in the sham-group the physical therapist palpated the reference point displayed on the block card. At the follow-up cue,'' GO'', the subject performed the index test, only this time during this movement the physical therapist only kept light contact with the dermis (upper layer of the skin) till the subject was back in starting position.

4. Skin Displacement condition orders

Different condition orders had been made **(Table 2)**. The index test and SKD condition that should be applied were shown on a block card **(Figure 9)** to the physical therapist concealed from the subject. Subsequently, the subject was instructed by the physical therapist which index test should be performed regarding one of the four pre-selected orders of testing **(Table 3)**.

Legends to the figures

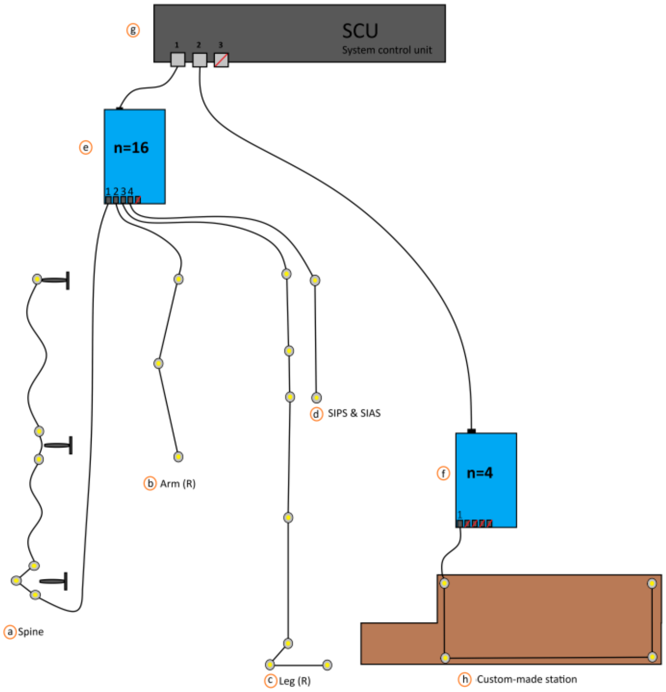

Figure 1| Connection process/ Abbreviations: CSU, system control unit; SIAS, Anterior superior sacroiliac joint; SIPS, posterior superior sacroiliac joint. **a,** spine; b, arm; **c,** leg; **d,** Illium; **e,** 1st strober; **f,** 2nd strober; **g,**SCU; **h,** custom-made station

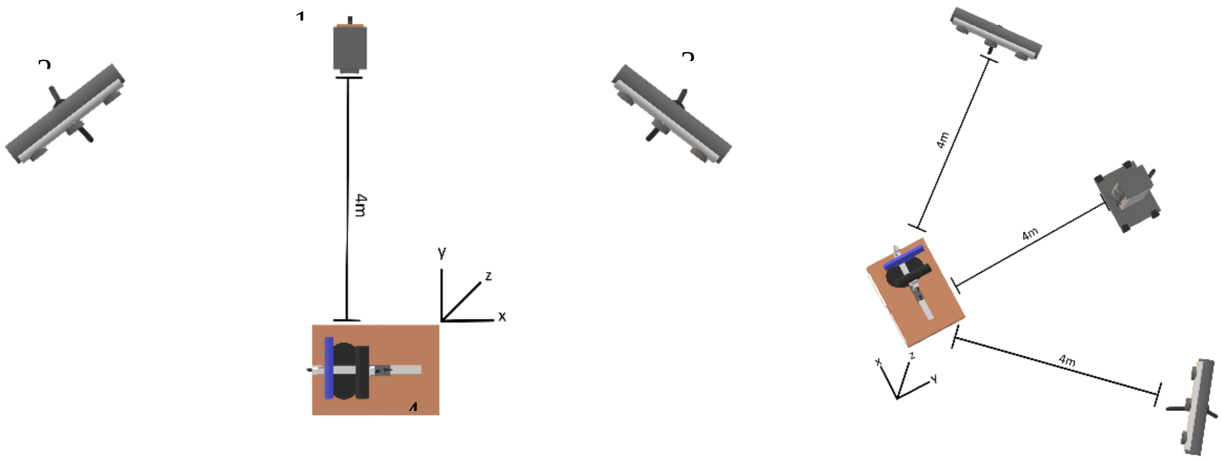

Figure 2| Camera & station set-up

1, Certus camera vertical; 2, Certus camera horizontal; 3, Certus camera; 4, horizontal side station

**1A**	**1B**	**2A**	**2B**
Practise session (10x extension and 10x flexion)	Practise session (10x extension and 10x flexion)	Practise session (10x extension and 10x flexion)	Practise session (10x extension and 10x flexion)
↓ 60 sec	↓ 60 sec	↓ 60 sec	↓ 60 sec
3 times baseline extension index test	3 times baseline extension index test	3 times baseline flexion index test	3 times baseline flexion index test
↓ 120 sec.	↓ 120 sec	↓ 120 sec	↓ 120 sec
Flexion + SKD LL3	Flexion + SKD RL3	Extension + SKD RL3	Extension +SKD RL3
↑↓ 30 sec	↑↓ 30 sec	↑↓ 30 sec	↑↓ 30 sec
Extension + SKD RL5	Extension + SKD LL3	Flexion + SKD LL5	Flexion + SKD LL5
↓ 180 sec.	↓ 180 sec	↓ 180 sec	↓ 180 sec
Flexion + SKD RL3	Flexion + SKD RL3	Extension + SKD LL3	Extension + SKD RL5
↑↓ 30 sec	↑↓ 30 sec	↑↓ 30 sec	↑↓ 30 sec

Extension + SKD LL5	Extension + SKD LL5	Flexion + RL5	Flexion + SKD LL3
↓ 10 min.	↓ 10 min	↓ 10 min	↓ 10 min
Cross-over to **2A**	Cross-over to **2B**	Cross-over to **1A**	Cross-over to **1B**

Figure 3| Flowchart SKD condition- order

– · → Are the 30 seconds rest periods between each executed index test (repeated 3 times)

⟶ Are the various inserted rest periods to wash out the carry-over effect

- SKD LL3, Skin Displacement to the left on location L3
- SKD RL3, Skin Displacement to the right on location L3
- SKD LL5, Skin Displacement to the left on location L5
- SKD RL5, Skin Displacement to the right on location L5

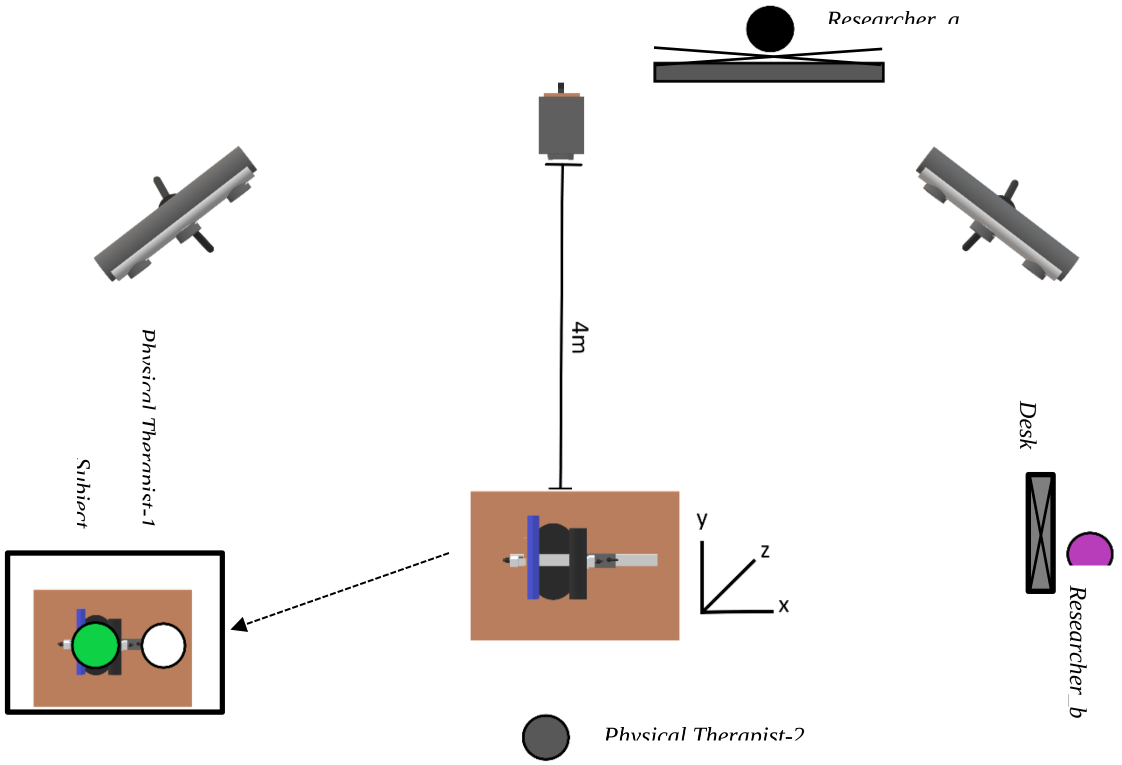

Figure 4| Camera, station set-up, and division task

Researcher_a: monitors computer and instructs the subject/PT

Researcher_b: shows the block cards, monitors the subject and timer, and counts out loud

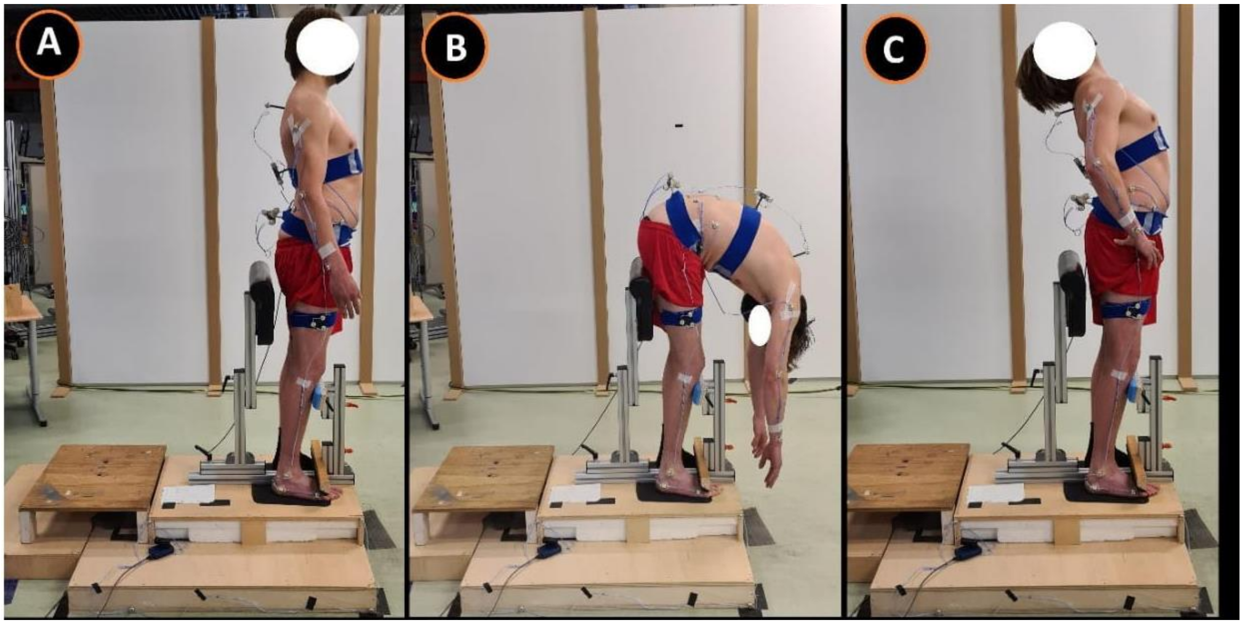

Figure 5| Index tests: baseline flexion and extension. The end ROM is monitored and counted out loudly by researcher_b. Each test is repeated 3 times separated from each other, see appendix II.

A. Neutral position; **B.** Trunk flexion; **C.** Trunk extension

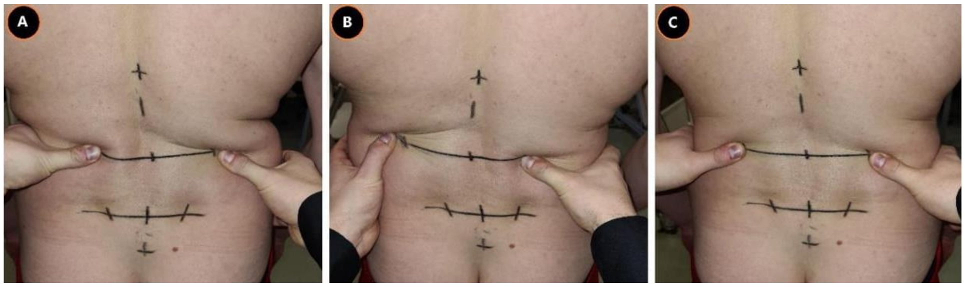

Figure 6| Skin Displacement. **A,** Skin displacement to the right; **B,** Skin displacement to the left; **C,** Skin displacement to the left

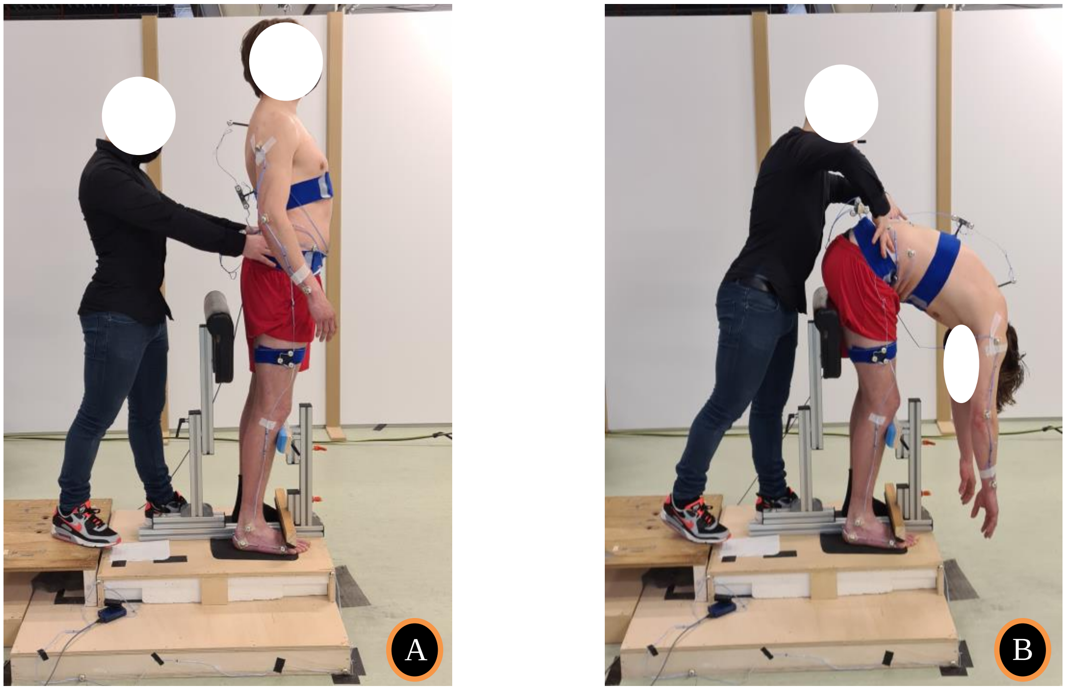

Figure 7| Index flexion test incl. SKD. **A,** Neutral start position, **B,** Spinopelvic-hip flexion

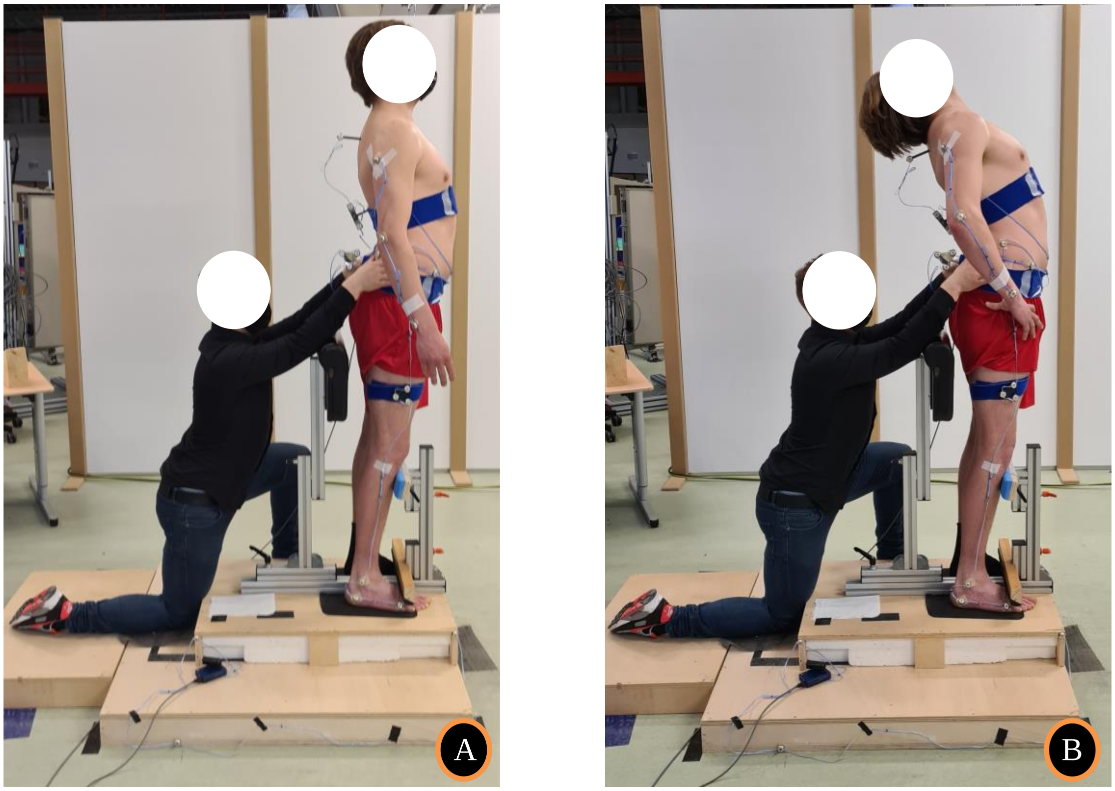

Figure 8| Index extension test incl. SKD. **A,** Neutral start position; **B,** Spinopelvic-hip flexion

Figure 9. Block card with conditions

**EXTENSION|SKD LEFT L3 (LL3)**

**EXTENSION|SKD RIGHT L3 (RL3)**

**EXTENSION|SKD LEFT L5 (LL5)**

**EXTENSION|SKD RIGHT L5 (RL5)**

**FLEXION|SKD LEFT L3 (LL3)**

**FLEXION|SKD RIGHT L3 (RL3)**

**FLEXION|SKD LEFT L5 (LL5)**

**FLEXION|SKD RIGHT L5 (RL5)**

<table>
<tr><th>Division task</th><th>Instructions</th></tr>
<tr><td>Pre-instruction physiotherapist</td><td>Oral instruction of the full assessment
Orally instruction of the specific movement, dependent on the randomization (a or b)
Trunk movement Execution:</td></tr>
<tr><td colspan="2">a) Trunk flexion (The movement is performed subject own controlled speed)</td></tr>
<tr><td>Instruction physiotherapist

pre- rehearsal</td><td>1. Start position: Instruct the subject to stand up straight, hanging arms, thumbs forward, feet parallel and contiguous, and gaze forward.
2. On cue: Bring your chin to your chest and then roll down from torso to lower back and then bend as far as you can while your hand reaches toward the station edge (marker 1) without forcing.
3. Researcher b counts loudly,’’1,2,3,4’’, and the subject comes back to start position.</td></tr>
<tr><td colspan="2">b) Trunk extension (The movement is performed subject own controlled speed)</td></tr>
<tr><td>Instruction physiotherapist

pre- rehearsal</td><td>1. Start position: Instruct the subject to stand up straight, hanging arms, thumbs forward, feet parallel and contiguous, and gaze forward.
2. On cue: Place your hands on your hips and extend your head then extend from torso to lower back and then extend as far as you can without forcing.
3. Researcher b counts loudly,’’1,2,3,4’’, and the subject comes back to start position.</td></tr>
<tr><td colspan="2">c) Execution (The movement is performed subject own controlled speed)</td></tr>
<tr><td>Instruction researchers of one full repetition</td><td>a. Subject and physiotherapist: stands in the start position
b. Researcher b: informs the physiotherapist which test should be performed utilizing block cards (Figure 9)
c. Researcher a: cues
d. Researcher a: remarks = ‘’10 seconds’’!
e. Physiotherapist: Palpation of the reference points displayed on the block card
f. Researcher a: ‘’camera set’’</td></tr>
</table>

	g. The physiotherapist performed the SKD as given by researcher b<br>h. Researcher a: Cue ‘‘GO’’!<br>• The physiotherapist performs the SKD maneuver and holds it under tension<br>• The subject performed the trunk motion.<br>i. Researcher b: monitors the subject’s end range of motion and counts loudly,’’1,2,3,4’’ in this end range monitored with a stopwatch.<br>j. Subject: after the 4 seconds the subject comes back to the start position.

Table 1| Summary subject instruction and division tasks

SKD condition	SKD order	Index test order
1A:	b,c ; a,d	Flexion -> extension
2A:	a,d; b,d	Extension -> Flexion
1B:	c,b; a,d	Flexion -> extension
2B:	a,d; c,b	Extension -> Flexion

Table 2| SKD maneuver condition. SKD conditions consist of Directions and Locations: a, Right SKD at level L3; b, Left SKD at level L3; c, Right SKD at level L5; d, Left SKD at level L5. SKD- and index test orders are mixed as displayed.

Table 3. Checklists different orders of testing

Checklist 1A								
	Warming- up			10 x flexion + 10 x extension				
60 seconds								
			Baseline ET 1st	30 seconds		1		
			Baseline ET 2nd	30 seconds		2		
			Baseline ET 3rd	30 seconds		3		
			120 sec					
	**Check box**	**SKD**	**Execution**	**Location**	**Direction**	**Remarks**		
						**Nr.**	**P1/P2**	
1.		**b**	Flexion 1st	L3	Left	4		
			30 seconds					
		**c**	Extension 1st	L5	Right	5		
			30 seconds					
2.		**b**	Flexion 2st	L3	Left	6		
			30 seconds					
		**c**	Extension 2nd	L5	Right	7		
			30 seconds					
3.		**b**	Flexion 3st	L3	Left	8		

			30 seconds					
		**c**	Extension 3rd	L5	Right	9		
			180 sec					
	**Check box**	**SKD**	**Execution**	**Location**	**Direction**	**Remarks**		
						**Nr.**	**P1/P2**	
1.		**a**	Flexion 1st	L3	Right	10		
			30 seconds					
		**d**	Extension 1st	L5	Left	11		
			30 seconds					
2.		**a**	Flexion 2st	L3	Right	12		
			30 seconds					
		**d**	Extension 2nd	L5	Left	13		
			30 seconds					
3.		**a**	Flexion 3st	L3	Right	14		
			30 seconds					
		**d**	Extension 3rd	L5	Left	15		

Checklist 2A								
	Warming- up			10 x flexion		10 x extension		
60 seconds								
			Baseline FL 1st	30 seconds		16		
			Baseline FL 2nd	30 seconds		17		
			Baseline FL 3rd	30 seconds		18		
			120 sec					
	**Check box**	**SKD**	**Execution**	**Location**	**Direction**	**Remarks**		
						**Nr.**	**P1/P2**	
1.		**a**	Extension 1st	L3	Right	19		
			30 seconds					
		**d**	Flexion 1st	L5	Left	20		
			30 seconds					
2.		**a**	Extension 2nd	L3	Right	21		
			30 seconds					
		**d**	Flexion 2nd	L5	Left	22		
			30 seconds					
3.		**a**	Extension 3rd	L3	Right	23		

			30 seconds					
		**d**	Flexion 3rd	L5	Left	24		
			180 sec					
	**Check box**	**SKD**	**Execution**	**Location**	**Direction**	**Remarks**		
						**Nr.**	**P1/P2**	
1.		**b**	Extension 1st	L3	Left	25		
			30 seconds					
		**c**	Flexion 1st	L5	Right	26		
			30 seconds					
2.		**b**	Extension 2nd	L3	Left	27		
			30 seconds					
		**c**	Flexion 2nd	L5	Right	28		
			30 seconds					
3.		**b**	Extension 3rd	L3	Left	29		
			30 seconds					
		**c**	Flexion 3rd	L5	Right	30		

Checklist 1B								
V	Warming- up			10 x extension		10 x flexion		
				60 seconds				
V			Baseline ET 1st	30 seconds		1		
V			Baseline ET 2nd	30 seconds		2		
V			Baseline ET 3rd			3		
V			120 sec					
	**Check box**	**SKD**	**Execution**	**Location**	**Direction**	**Remarks**		
						**Nr.**	**P1/P2**	
1.	V	**c**	Flexion 1st	L5	Right	4		
	V		30 seconds					
	V	**b**	Extension 1st	L3	Left	6		
	V		30 seconds					
2.	V	**c**	Flexion 2nd	L5	Right	7		
	V		30 seconds					
	V	**b**	Extension 2nd	L3	Left	8		
	V		30 seconds					
3.	V	**c**	Flexion 3rd	L5	Right	9		

	v		30 seconds					
	v	**b**	Extension 3rd	L3	Left	10		
	V		180 sec					
	**Check box**	**SKD**	**Execution**	**Location**	**Direction**	**Remarks**		
						**Nr.**	**P1/P2**	
1.	V	**a**	Flexion 1st	L3	Right	11		
	V		30 seconds					
	V	**d**	Extension 1st	L5	Left	12		
	V		30 seconds					
2.	V	**a**	Flexion 2nd	L3	Right	13		
	V		30 seconds					
	V	**d**	Extension 2nd	L5	Left	14		
	V		30 seconds					
3.	V	**a**	Flexion 3rd	L3	Right	15		
	V		30 seconds					
	V	**d**	Extension 3rd	L5	Left	16		
Break 10 minutes & cross-over 2B								

Checklist 2B								
V	Warming- up			10 x flexion		10 x extension		
60 seconds								
V			Baseline FL 1st	30 seconds		17		
V			Baseline FL 2nd	30 seconds		18		
V			Baseline FL 3rd			19		
V			120 sec					
	**Check box**	**SKD**	**Execution**	**Location**	**Direction**	**Remarks**		
						**Nr.**	**P1/P2**	
1.	V	**a**	Extension 1st	L3	Right	20		
	V		30 seconds					
	V	**d**	Flexion 1st	L5	Left	21		
	V		30 seconds					
2.	V	**a**	Extension 2st	L3	Right	22		
	V		30 seconds					
	V	**d**	Flexion 2nd	L5	Left	23		
	V		30 seconds					
3.	V	**a**	Extension 3st	L3	Right	24		

	V		30 seconds					
	V	**d**	Flexion 3rd	L5	Left	25		
	V		180 sec					
	**Check box**	**block**	**Execution**	**Location**	**Direction**	**Remarks**		
						**Nr.**	**P1/P2**	
1.	V	**c**	Extension 1st	L5	Right	26		
	V		30 seconds					
	V	**b**	Flexion 1st	L3	Left	27		
	V		30 seconds					
2.	V	**c**	Extension 2st	L5	Right	28		
	V		30 seconds					
	V	**b**	Flexion 2st	L3	Left	29		
	V		30 seconds					
3.	V	**c**	Extension 3st	L5	Right	30		
	V		30 seconds					
	V	**b**	Flexion 3st	L3	Left	31		

5. MATLAB script

Figure 10. Matlab script

```
%% Loading data
%% ROM baseline
disp ('INDEX test results')
[x1,z1,y1] = readndf('TN00000x1.ndf');
[x2,z2,y2] = readndf('TN00000x2.ndf');
[x3,z3,y3] = readndf('TN00000x3.ndf');

%% ROM + SKD RL3
% disp ('SKD-condition')
% [x1,z1,y1] = readndf('TN00000x1.ndf');
% [x2,z2,y2] = readndf('TN00000x2.ndf');
% [x3,z3,y3] = readndf('TN00000x3.ndf');
% load('.mat')
% load('ICC.mat')

%% time domain
Fs = 100;
dt = 1/Fs;
N = length (x1);
k = [0:N-1];
t = k*dt;

%% Markers of the back: (Cluster) 1=S2,2=SC,3=S1, (Twin)4= T11, 5 = T9, (Single)6 = T4
%% Cluster legg:  11= craniaal 12= posterior, 13= anterior
%% Illium = 9 SIAS = 17 SIPS=18
%% FFD marker: 10= wrist
%% Transformation markers in row (!) 1:S2, 2:SC, 3:S1, 4:T11, 5:T9, 6:T4, 7:pols, 8:Illium mediaal, BC:9, BP:10, BA:11, SIAS:12, SIPS:13, station caudaal 14, station 15 craniaal
x1 = x1(:, [1:6,9:13,17:18,21:22]);
y1 = y1(:, [1:6,9:13,17:18,21:22]);
x2 = x2(:, [1:6,9:13,17:18,21:22]);
y2 = y2(:, [1:6,9:13,17:18,21:22]);
x3 = x3(:, [1:6,9:13,17:18,21:22]);
y3 = y3(:, [1:6,9:13,17:18,21:22]);

%% Marker plot x
figure (1)
subplot (2,1,1)
hold on
for i = 1:11
```

```
 plot(x1(:,i),'r'); hold on
 plot(x2(:,i),'k'); hold on
 plot(x3(:,i),'g'); hold on
end

figure (1)
subplot (2,1,2)
hold on
for i = 1:11
plot(y1(:,i),'r'); hold on
 plot(y2(:,i),'k'); hold on
 plot(y3(:,i),'g'); hold on
end


%% Interpolation
for i = 1:11
 x1 = fillmissing(x1,'linear');
 x2 = fillmissing(x2,'linear');
 x3 = fillmissing(x3,'linear');
end

for i = 1:11
 y1 = fillmissing(y1,'linear');
 y2 = fillmissing(y2,'linear');
 y3 = fillmissing(y3,'linear');
end

for i = 1:11
 z1 = fillmissing(z1,'linear');
 z2 = fillmissing(z2,'linear');
 z3 = fillmissing(z3,'linear');
end

%% Marker plot x2
figure (2)
subplot (2,1,1)
hold on
for i = 1:11
 plot(x1(:,i),'r'); hold on
 plot(x2(:,i),'k'); hold on
 plot(x3(:,i),'g'); hold on
end
```

```
figure (2)
subplot (2,1,2)
hold on
for i = 1:11
plot(y1(:,i),'r'); hold on
 plot(y2(:,i),'k'); hold on
 plot(y3(:,i),'g'); hold on
end

%% Filtering butter has 3 inputs 4th order
NF=4; % 4th order. 1st
fc= 5; % var for cutoff frequency  (low/high pass). 2nd
Wn=fc/(Fs/2); %%cutoff frequency (pass filt/ (fs/2). 2nd
[B,A]=butter(4,0.1);
FiltSigna1x=filter(B,A,x1);
FiltSigna2x=filter(B,A,x2);
FiltSigna3x=filter(B,A,x3);

FiltSigna1y=filter(B,A,y1);
FiltSigna2y=filter(B,A,y2);
FiltSigna3y=filter(B,A,y3);

FiltSigna1z=filter(B,A,z1);
FiltSigna2z=filter(B,A,z2);
FiltSigna3z=filter(B,A,z3);

%% Plot filter signal
%% figure
figure (3)
hold on
for i= 1:11
 grid on, hold on, subplot (5,2,1), plot (t,filter(B,A,x1(:,i)));
 grid on, hold on, subplot (5,2,3), plot (t,filter(B,A,x2(:,i)));
 grid on, hold on, subplot (5,2,5), plot (t,filter(B,A,x3(:,i)));
 xlabel('time (in seconds)');
 ylabel('signal');
 hold on
end

for i= 1:11
 grid on, hold on, subplot (5,2,2), plot (t,filter(B,A,y1(:,i)));
 grid on, hold on, subplot (5,2,4), plot (t,filter(B,A,y2(:,i)));
 grid on, hold on, subplot (5,2,6), plot (t,filter(B,A,y3(:,i)));
 xlabel('time (in seconds)');
```

```
 ylabel('signal');
 hold on
end

figure (4)
hold on
for i= 1:11
 grid on, hold on, subplot (5,2,1), plot (filter(B,A,x1(:,i)));
 grid on, hold on, subplot (5,2,2), plot (x1 (:,i),'-r');
 grid on, hold on, subplot (5,2,3), plot (filter(B,A,x2(:,i)));
 grid on, hold on, subplot (5,2,4), plot (x2 (:,i),'-r');
 grid on, hold on, subplot (5,2,5), plot (filter(B,A,x3(:,i)));
 grid on, hold on, subplot (5,2,6), plot (x3 (:,i),'-r');
 hold on
 title('butter raw')
 xlabel('1000(hz)');
 ylabel('signal');
end

figure (5)
hold on
for i= 1:11
 grid on, hold on, subplot (5,2,1), plot (filter(B,A,y1(:,i)));
 grid on, hold on, subplot (5,2,2), plot (y1 (:,i),'-r');
 grid on, hold on, subplot (5,2,3), plot (filter(B,A,y2(:,i)));
 grid on, hold on, subplot (5,2,4), plot (y2 (:,i),'-r');
 grid on, hold on, subplot (5,2,5), plot (filter(B,A,y3(:,i)));
 hold on
 title('butter raw')
 xlabel('1000(hz)');
 ylabel('signal');
end

%% reorganize
%% rewrite filter signal
x1= FiltSigna1x;
x2= FiltSigna2x;
x3= FiltSigna3x;


y1= FiltSigna1y;
y2= FiltSigna2y;
y3= FiltSigna3y;
```

```
z1= FiltSigna1z;
z2= FiltSigna2z;
z3= FiltSigna3z;

%% CAB angles
%% Sacrum cluster
S1_1 (:,1) = x1 (:,3); S1_2 (:,1) = x2 (:,3); S1_3 (:,1) = x3 (:,3);
S1_1 (:,2) = y3 (:,3); S1_2 (:,2) = y1 (:,3); S1_3 (:,2) = y3 (:,3);
S2_1 (:,1) = x1 (:,1); S2_2 (:,1) = x2 (:,1); S2_3 (:,1) = x3 (:,1);
S2_1 (:,2) = y1 (:,1); S2_2 (:,2) = y1 (:,1); S2_3 (:,2) = y3 (:,1);
SC_1 (:,1) = x1 (:,2); SC_2 (:,1) = x2 (:,2); SC_3 (:,1) = x3 (:,2);
SC_1 (:,2) = y1 (:,2); SC_2 (:,2) = y1 (:,2); SC_3 (:,2) = y3 (:,2);

%% Centroid
Sacrum_x1 = (S1_1 (:,1) + S2_1 (:,1) + SC_1 (:,1))/3; Sacrum_x2 = (S1_2 (:,1) + S2_2 (:,1) + SC_2 (:,1))/3; Sacrum_x3 = (S1_3 (:,1) + S2_3 (:,1) +
SC_3 (:,1))/3;
Sacrum_y1 = (S1_1 (:,2) + S2_1 (:,2) + SC_1 (:,2))/3; Sacrum_y2 = (S1_2 (:,2) + S2_2 (:,2) + SC_2 (:,2))/3; Sacrum_y3 = (S1_3 (:,2) + S2_3 (:,2) +
SC_3 (:,2))/3;

%% SACRUM
Sacrum1 (:,1) = (Sacrum_x1); Sacrum2 (:,1) = (Sacrum_x2); Sacrum3 (:,1) = (Sacrum_x3);
Sacrum1 (:,2) = (Sacrum_y1); Sacrum2 (:,2) = (Sacrum_y2); Sacrum3 (:,2) = (Sacrum_y3);


il_1 (:,1) = x1 (:,8); il_2 (:,1) = x2 (:,8); il_3 (:,1) = x3 (:,8);
il_1 (:,2) = y1 (:,8); il_2 (:,2) = y2 (:,8); il_3 (:,2) = y3 (:,8);
SIPS_1 (:,1) = x1 (:,12); SIPS_2 (:,1) = x2 (:,12); SIPS_3 (:,1) = x3 (:,12);
SIPS_1 (:,2) = y1 (:,12); SIPS_2 (:,2) = y2 (:,12); SIPS_3 (:,2) = y3 (:,12);
SIAS_1 (:,1) = x1 (:,12); SIAS_2 (:,1) = x2 (:,12); SIAS_3 (:,1) = x3 (:,12);
SIAS_1 (:,2) = y1 (:,12); SIAS_2 (:,2) = y2 (:,12); SIAS_3 (:,2) = y3 (:,12);

%% centroid pelvic
Pelvic1 = (Sacrum1 + il_1)/2;Pelvic2 = (Sacrum2 + il_2)/2;Pelvic3 = (Sacrum3 + il_3)/2;

%% hip (cluster marker
BC_1 (:,1) = x1 (:,9); BC_2 (:,1) = x2 (:,9); BC_3 (:,1) = x3 (:,9);
BC_1 (:,2) = y1 (:,9); BC_2 (:,2) = y2 (:,9); BC_3 (:,2) = y3 (:,9);
BP_1 (:,1) = x1 (:,10); BP_2 (:,1) = x2 (:,10); BP_3 (:,1) = x3 (:,10);
BP_1 (:,2) = y1 (:,10); BP_2 (:,2) = y2 (:,10); BP_3 (:,2) = y3 (:,10);
BA_1 (:,1) = x1 (:,11); BA_2 (:,1) = x2 (:,11); BA_3 (:,1) = x3 (:,11);
BA_1 (:,2) = y1 (:,11); BA_2 (:,2) = y2 (:,11); BA_3 (:,2) = y3 (:,11);

% centroid
Legg_x1 = (BC_1 (:,1) + BP_1 (:,1) + BA_1 (:,1))/3;Legg_x2 = (BC_2 (:,1) + BP_2 (:,1) + BA_2 (:,1))/3; Legg_x3 = (BC_3 (:,1) + BP_3 (:,1) + BA_3
(:,1))/3;
```

```
Legg_y1 = (BC_1 (:,1) + BP_1 (:,1) + BA_1 (:,1))/3;Legg_y2 = (BC_2 (:,1) + BP_2 (:,1) + BA_2 (:,1))/3; Legg_y3 = (BC_3 (:,1) + BP_3 (:,1) + BA_3 (:,1))/3;



% upper legg
Legg1 (:,1) = (Legg_x1); Legg2 (:,1) = (Legg_x2); Legg3 (:,1) = (Legg_x3);
Legg1 (:,2) = (Legg_y1); Legg2 (:,2) = (Legg_y2); Legg3 (:,2) = (Legg_y3);

%% spine
T11_1 (:,1) = x1 (:,4); T11_2 (:,1) = x2 (:,4); T11_3 (:,1) = x3 (:,4);
T11_1 (:,2) = y1 (:,4); T11_2 (:,2) = y2 (:,4); T11_3 (:,2) = y3 (:,4);
T9_1 (:,1) = x1 (:,5); T9_2 (:,1) = x2 (:,5); T9_3 (:,1) = x3 (:,5);
T9_1 (:,2) = y1 (:,5); T9_2 (:,2) = y2 (:,5); T9_3 (:,2) = y3 (:,5);
T4_1 (:,1) = x1 (:,6); T4_2 (:,1) = x2 (:,6); T4_3 (:,1) = x3 (:,6);
T4_1 (:,2) = y1 (:,6); T4_2 (:,2) = y2 (:,6); T4_3 (:,2) = y3 (:,6);

%% TLO centroid
TLO_x1 = T11_1 (:,1) + T9_1 (:,1)/2; TLO_x2 = T11_2 (:,1) + T9_2 (:,1)/2; TLO_x3 = T11_3 (:,1) + T9_3 (:,1)/2;
TLO_y1 = T11_1(:,2)+T9_1 (:,2)/2; TLO_y2 = T11_2 (:,2) + T9_2 (:,2)/2; TLO_y3 = T11_3 (:,2) + T9_3 (:,2)/2;
TLO1 (:,1) = (TLO_x1); TLO2 (:,1) = (TLO_x2); TLO3 (:,1) = (TLO_x3);
TLO1 (:,2) = (TLO_y1); TLO2 (:,2) = (TLO_y2); TLO3 (:,2) = (TLO_y3);

%% plot the required markers check crosstalk
figure (6)
for i= 1:11
 grid on, hold on, subplot (5,1,1), plot (Legg1);
 grid on, hold on, subplot (5,1,2), plot (Sacrum1);
 grid on, hold on, subplot (5,1,3), plot (T11_1);
 grid on, hold on, subplot (5,1,4), plot (T9_1);
 grid on, hold on, subplot (5,1,5), plot (T4_1);
 xlabel('time (in seconds)');
 ylabel('signal');
end



%%%%%%%%%%%
%% HROM%%%%
%%%%%%%%%%%
%% The sagittal hip angle CAB !! proces (B:Illium, A:Sacrum, C: Legg)
x1=[Legg1(:,1),Sacrum1(:,1),il_1(:,1)];
x2=[Legg2(:,1),Sacrum2(:,1),il_2(:,1)];
x3=[Legg3(:,1),Sacrum3(:,1),il_3(:,1)];
```

```
y1=[Legg1(:,2),Sacrum1(:,2),il_1(:,2)];
y2=[Legg2(:,2),Sacrum2(:,2),il_2(:,2)];
y3=[Legg3(:,2),Sacrum3(:,2),il_3(:,2)];
%% fictive segements
% calculate the angle between the two lines
fiseg1(:,1)= atan2d( (y1(:,2)-y1(:,1)),(x1(:,2)-x1(:,1)) );
fiseg2(:,1)= atan2d( (y2(:,2)-y2(:,1)),(x2(:,2)-x2(:,1)) );
fiseg3(:,1)= atan2d( (y3(:,2)-y3(:,1)),(x3(:,2)-x3(:,1)) );


fiseg1(:,2)= atan2d( (y1(:,3)-y1(:,2)),(x1(:,3)-x1(:,2)) );
fiseg2(:,2)= atan2d( (y2(:,3)-y2(:,2)),(x2(:,3)-x2(:,2)) );
fiseg3(:,2)= atan2d( (y3(:,3)-y3(:,2)),(x3(:,3)-x3(:,2)) );

%% calculate tangential angle (the angle between the lines)
HROM1= fiseg1(:,2)-fiseg1(:,1); HROM2= fiseg2(:,2)-fiseg2(:,1); HROM3= fiseg3(:,2)-fiseg3(:,1);

%% plot HROM
figure (7)
subplot (5,1,1), plot (HROM1,'--k'); grid on, hold on;
subplot (5,1,2), plot (HROM2,'--r'); grid on, hold on;
subplot (5,1,3), plot (HROM3,'--g'); grid on, hold on;
hold on, grid on;
hold on, xlabel 'frame'; ylabel 'hip angle [degrees]';

%% multi
figure (8)
plot (HROM1,'--k'); grid on, hold on;
plot (HROM2,'--r'); grid on, hold on;
plot (HROM3,'--g'); grid on, hold on;

hold on, grid on;
hold on, xlabel 'frame'; ylabel 'hip angle [degrees]';

figure (8)
plot (HROM1,'--k'); grid on, hold on;
plot (HROM2,'--r'); grid on, hold on;
plot (HROM3,'--g'); grid on, hold on;
% plot (HROM4,'--c'); grid on, hold on;
% plot (HROM5,'--y'); grid on, hold on;
hold on, grid on;
title 'HIP angle during  [inverse]'
hold on, xlabel 'frame'; ylabel 'hip angle [degrees]';
```

```
%% mean (excluded 2 outliers ROM1= zwart, ROM2= rood, ROM3= groen, ROM4= cayen, ROM5= blauw
%% Time shift
Px1 = ginput(2);Px2 = ginput(2);Px3 = ginput(2);
Px1= round(Px1); Px2= round(Px2);Px3= round(Px3);
HROM1= HROM1(Px1(1):Px1(2));
HROM2= HROM2(Px2(1):Px2(2));
HROM3= HROM3(Px3(1):Px3(2));

figure(10)
grid on,hold on, plot (HROM1,'--k'); grid on, hold on; plot (HROM2,'--r'); grid on, hold on; plot (HROM3,'--g'); grid on, hold on;

%% ginput
figure (10)
idx = ginput(2);
idx= round(idx);
NHROM1= HROM1(idx(1):idx(2));
NHROM2= HROM2(idx(1):idx(2));
NHROM3= HROM3(idx(1):idx(2));

Test1_HROM= mean (NHROM1);
Test2_HROM= mean (NHROM2);
Test3_HROM= mean (NHROM3);

%% mean best fit time
HROMmean = (NHROM1) + (NHROM2) +(NHROM3);
HROMmean = (HROMmean)/3;
figure (11)
subplot (2,1,1), plot(HROMmean,'--k'); hold on; plot (HROMmean,'*'); hold on;
title 'HIP angle during  [inverse]'
hold on, xlabel 'frame'; ylabel 'hip angle [degrees]';
hold on
figure (11)
subplot (2,1,2), boxplot (HROMmean);

%% index
END_HROMmean= mean (HROMmean);
SD_HROMmean = std (HROMmean)*2;

%% baseline & end ROM is saved in xls
disp ('HROM mean')
END_HROMmean
```

```
%%%%%%%%%%%%%%%%
%% Lumbar ROM %%
%%%%%%%%%%%%%%%%
%% The sagittal lumbar angle CAB !! proces (B:legg, A:sacrum, C: TLO)
x1b=[Legg1(:,1),Sacrum1(:,1),TLO1(:,1)];
x2b=[Legg2(:,1),Sacrum2(:,1),TLO2(:,1)];
x3b=[Legg3(:,1),Sacrum3(:,1),TLO3(:,1)];

y1b=[Legg1(:,2),Sacrum1(:,2),TLO1(:,2)];
y2b=[Legg2(:,2),Sacrum2(:,2),TLO2(:,2)];
y3b=[Legg3(:,2),Sacrum3(:,2),TLO3(:,2)];

%% fictive segements opposite = y and adjacent = x
fiseg1b(:,1)= atan2d( (y1b(:,2)-y1b(:,1)),(x1b(:,2)-x1b(:,1)) ); %IL-TLO
fiseg2b(:,1)= atan2d( (y2b(:,2)-y2b(:,1)),(x2b(:,2)-x2b(:,1)) );
fiseg3b(:,1)= atan2d( (y3b(:,2)-y3b(:,1)),(x3b(:,2)-x3b(:,1)) );


fiseg1b(:,2)= atan2d( (y1b(:,3)-y1b(:,2)),(x1b(:,3)-x1b(:,2)) ); % IL-Sacrum
fiseg2b(:,2)= atan2d( (y2b(:,3)-y2b(:,2)),(x2b(:,3)-x2b(:,2)) );
fiseg3b(:,2)= atan2d( (y3b(:,3)-y3b(:,2)),(x3b(:,3)-x3b(:,2)) );



%% (IL-TLO)- (IL-Sacrum)
% calculate tangential angle (the angle between the lines)
LROM1= fiseg1b(:,2)-fiseg1b(:,1); LROM2= fiseg2b(:,2)-fiseg2b(:,1); LROM3= fiseg3b(:,2)-fiseg3b(:,1);

%% plot LROM
figure (12)
plot (LROM1,'--k'); grid on, hold on;
plot (LROM2,'--r'); grid on, hold on;
plot (LROM3,'--g'); grid on, hold on;
hold on, grid on;
hold on, xlabel 'frame'; ylabel 'LUMBAR angle [degrees]';

%% mean (excluded 2 outliers ROM1= zwart, ROM2= rood, ROM3= groen, ROM4= cayen, ROM5= blauw
%% Time shift
Px1 = ginput(2);Px2 = ginput(2);Px3 = ginput(2);
Px1= round(Px1); Px2= round(Px2);Px3= round(Px3);
LROM1= LROM1(Px1(1):Px1(2));
LROM2= LROM2(Px2(1):Px2(2));
```

```
LROM3= LROM3(Px3(1):Px3(2));

figure(13)
grid on,hold on, plot (LROM1,'--k'); grid on, hold on; plot (LROM2,'--r'); grid on, hold on; plot (LROM3,'--g'); grid on, hold on;

%% ginput
figure (13)
idx = ginput(2);
idx= round(idx);
NLROM1= LROM1(idx(1):idx(2));
NLROM2= LROM2(idx(1):idx(2));
NLROM3= LROM3(idx(1):idx(2));
%%
Test1_LROM= mean (NLROM1);
Test2_LROM= mean (NLROM2);
Test3_LROM= mean (NLROM3);

%% mean best fit time
LROMmean = (NLROM1) + (NLROM2) +(NLROM3);
LROMmean = (LROMmean)/3;
figure (14)
subplot (2,1,1), plot(LROMmean,'--k'); hold on; plot (LROMmean,'*'); hold on;
title 'LUMBAR angle during  [inverse]'
hold on, xlabel 'frame'; ylabel 'LUMBAR angle [degrees]';
hold on
figure (14)
subplot (2,1,2), boxplot (LROMmean);

%% index
END_LROMmean= mean (LROMmean);
SD_LROMmean = std (LROMmean)*2;

%% baseline & end ROM
disp ('LROM mean')
END_LROMmean



%%%%%%%%%%
%% TROM%%%
%%%%%%%%%%
%% The sagittal Trunk angle CAB !! proces (B:Legg, A:sacrum, C: T4)
% new x and y
```

```
x1b=[Legg1(:,1),Sacrum1(:,1),T4_1(:,1)];
x2b=[Legg2(:,1),Sacrum2(:,1),T4_2(:,1)];
x3b=[Legg3(:,1),Sacrum3(:,1),T4_3(:,1)];

y1b=[Legg1(:,2),Sacrum1(:,2),T4_1(:,2)];
y2b=[Legg2(:,2),Sacrum2(:,2),T4_2(:,2)];
y3b=[Legg3(:,2),Sacrum3(:,2),T4_3(:,2)];

%% fictive segements opposite = y and adjacent = x
fiseg1b(:,1)= atan2d( (y1b(:,2)-y1b(:,1)),(x1b(:,2)-x1b(:,1)) ); %IL-TLO
fiseg2b(:,1)= atan2d( (y2b(:,2)-y2b(:,1)),(x2b(:,2)-x2b(:,1)) );
fiseg3b(:,1)= atan2d( (y3b(:,2)-y3b(:,1)),(x3b(:,2)-x3b(:,1)) );


fiseg1b(:,2)= atan2d( (y1b(:,3)-y1b(:,2)),(x1b(:,3)-x1b(:,2)) ); % IL-Sacrum
fiseg2b(:,2)= atan2d( (y2b(:,3)-y2b(:,2)),(x2b(:,3)-x2b(:,2)) );
fiseg3b(:,2)= atan2d( (y3b(:,3)-y3b(:,2)),(x3b(:,3)-x3b(:,2)) );


%% (IL-TLO)- (IL-Sacrum)
% calculate tangential angle (the angle between the lines)
TROM1= fiseg1b(:,2)-fiseg1b(:,1); TROM2= fiseg2b(:,2)-fiseg2b(:,1); TROM3= fiseg3b(:,2)-fiseg3b(:,1);

%% plot TROM
figure (15)
plot (TROM1,'--k'); grid on, hold on;
plot (TROM2,'--r'); grid on, hold on;
plot (TROM3,'--g'); grid on, hold on;
hold on, grid on;
hold on, xlabel 'frame'; ylabel 'TRUNK angle [degrees]';

%% mean (excluded 2 outliers ROM1= zwart, ROM2= rood, ROM3= groen, ROM4= cayen, ROM5= blauw
%% Time shift
Px1 = ginput(2);Px2 = ginput(2);Px3 = ginput(2);
Px1= round(Px1); Px2= round(Px2);Px3= round(Px3);
TROM1= TROM1(Px1(1):Px1(2));
TROM2= TROM2(Px2(1):Px2(2));
TROM3= TROM3(Px3(1):Px3(2));

figure(16)
grid on, hold on, plot (TROM1,'--k'); grid on, hold on; plot (TROM2,'--r'); grid on, hold on; plot (TROM3,'--g'); grid on, hold on;

%% ginput
figure (16)
```

```
idx = ginput(2);
idx= round(idx);
NTROM1= TROM1(idx(1):idx(2));
NTROM2= TROM2(idx(1):idx(2));
NTROM3= TROM3(idx(1):idx(2));

Test1_TROM= mean (NTROM1);
Test2_TROM= mean (NTROM2);
Test3_TROM= mean (NTROM3);

%% mean best fit time
TROMmean = (NTROM1) + (NTROM2) +(NTROM3);
TROMmean = (TROMmean)/3;
figure (17)
subplot (2,1,1), plot(TROMmean,'--k'); hold on; plot (TROMmean,'*'); hold on;
title 'Total TRUNK angle during  [inverse]'
hold on, xlabel 'frame'; ylabel 'Total TRUNK angle [degrees]';
% hold on, set (gca,'Yticklabel', [-52,-50, -45, -40,-35, -30, -24, - 25]);
hold on
figure (17)
subplot (2,1,2), boxplot (TROMmean);

%% input
END_TROMmean= mean (TROMmean);
% SD_TROMmean = std (TROMmean)*2;



%% baseline & end ROM
disp ('TROM mean')
END_TROMmean


%% for Save file
% ROM_SKD (:,7)= END_HROMmean;
% ROM_SKD (:,8)= END_LROMmean;
% ROM_SKD (:,9)= END_TROMmean;

%% Inter class rel.save
% ROM_ICC (:,19)= Test1_HROM;
% ROM_ICC (:,20)= Test2_HROM;
% ROM_ICC (:,21)= Test3_HROM;

%% Transform into xlsx file
```

```
% save('ROM.mat','flexion_SKD')
% save('ROM_ICC.mat','flexion_ICC')
% xlswrite('ROM.xlsx',flexion_SKD);
% xlswrite('ROM_ICC.xlsx',flexion_ICC);
xlswrite('TROMmean.xlsx',TROMmean);
```

de las Penas, C.P. (2019). "Can manual therapy focus on the thoracolumbar fasca? A clinical evidence-based ultrasonic exploration.", in: *world congress on low back and pelvic girdle pain 2019.* (ed.) V.a. Danneels. (Antwerp).

Huijing, P.A., and Baan, G.C. (2003). Myofascial force transmission: muscle relative position and length determine agonist and synergist muscle force. *Journal of applied physiology* 94(3)**,** 1092-1107.

Maas, H. (2019). Significance of epimuscular myofascial force transmission under passive muscle conditions. *Journal of Applied Physiology* 126(5)**,** 1465-1473.

Noten, K. (2021). *The Dynamic ArthroMyofascial Translation® Test (DAMT®Test). (4xT®Method the ArthroMyofascial Therapy: Chapter 2)* [Online]. Available: https://osf.io/52ze7 [Accessed].

Tu, S.J., Woledge, R.C., and Morrissey, D. (2016). Does 'Kinesio tape'alter thoracolumbar fascia movement during lumbar flexion? An observational laboratory study. *Journal of bodywork and movement therapies* 20(4)**,** 898-905.

Wang, C.K., Fang, Y.H.D., Lin, L.C., Lin, C.F., Kuo, L.C., Chiu, F.M., et al. (2019). Magnetic Resonance Elastography in the Assessment of Acute Effects of Kinesio Taping on Lumbar Paraspinal Muscles. *Journal of Magnetic Resonance Imaging* 49(4)**,** 1039-1045. doi: 10.1002/jmri.26281.

Wong, K.-K., Chai, H.-M., Chen, Y.-J., Wang, C.-L., Shau, Y.-W., and Wang, S.-F. (2017). Mechanical deformation of posterior thoracolumbar fascia after myofascial release in healthy men: a study of dynamic ultrasound imaging. *Musculoskeletal Science and Practice* 27**,** 124-130.
